# MicroRNA Delivery by Graphene-Based Complexes into Glioblastoma Cells

**DOI:** 10.3390/molecules26195804

**Published:** 2021-09-25

**Authors:** Marta Kutwin, Malwina Ewa Sosnowska, Barbara Strojny-Cieślak, Slawomir Jaworski, Maciej Trzaskowski, Mateusz Wierzbicki, Andre Chwalibog, Ewa Sawosz

**Affiliations:** 1 Department of Nanobiotechnology, Institute of Biology, Warsaw University of Life Sciences, 02-786 Warsaw, Poland; malwina_sosnowska@sggw.edu.pl (M.E.S.); barbara_strojny@sggw.edu.pl (B.S.-C.); slawomir_jaworski@sggw.pl (S.J.); mateusz_wierzbicki@sggw.edu.pl (M.W.); ewa_sawosz@sggw.edu.pl (E.S.); 2 Centre for Advanced Materials and Technologies CEZAMAT, Warsaw University of Technology, 02-822 Warsaw, Poland; m.trzaskowski@cezamat.pl; 3 Department of Veterinary and Animal Sciences, Faculty of Health and Medical Sciences, University of Copenhagen, 1870 Frederiksberg, Denmark; ach@sund.ku.dk

**Keywords:** cancer, glioblastoma, microRNA, graphene, complexes

## Abstract

Glioblastoma (GBM) is the most common primary and aggressive tumour in brain cancer. Novel therapies, despite achievements in chemotherapy, radiation and surgical techniques, are needed to improve the treatment of GBM tumours and extend patients’ survival. Gene delivery therapy mostly uses the viral vector, which causes serious adverse events in gene therapy. Graphene-based complexes can reduce the potential side effect of viral carries, with high efficiency of microRNA (miRNA) or antisense miRNA delivery to GBM cells. The objective of this study was to use graphene-based complexes to induce deregulation of miRNA level in GBM cancer cells and to regulate the selected gene expression involved in apoptosis. The complexes were characterised by Fourier transform infrared spectroscopy (FTIR), scanning transmission electron microscopy and zeta potential. The efficiency of miRNA delivery to the cancer cells was analysed by flow cytometry. The effect of the anticancer activity of graphene-based complexes functionalised by the miRNA sequence was analysed using 2,3-*bis*-(2-methoxy-4-nitro-5-sulfophenyl)-2*H*-tetrazolium-5-carboxyanilide salt (XTT) assays at the gene expression level. The results partly explain the mechanisms of miRNA deregulation stress, which is affected by graphene-based complexes together with the forced transport of mimic miR-124, miR-137 and antisense miR-21, -221 and -222 as an anticancer supportive therapy.

## 1. Background

Nanobiotechnology is one of the fastest-developing fields of science. The advantages of using nanoachievements are based on combining chemistry, physics and biotechnology. Nanobiotechnology in medicine has the potential to develop new types of molecules for the diagnosis or treatment of different types of diseases, including cancer. Since graphene nanosheets were first successfully isolated in 2004, graphene has been constantly and intensively studied because of this unique physiochemical property [1]. Graphene has a high active surface–mass volume ratio and strong C-C covalent bonds which extremely increase its mechanical resistance and exceptional thermal and electrical conductivity [2]. For the biomedical application, the 2D structure of graphene is beneficial because of its specific and monolayer surface area, which can be successfully loaded and interact with biological molecules or chemical compounds [3]. The free π electrons on the horizontal surface of graphene enable loading onto the surface through electrophilic reaction with biomolecules, but graphene can also absorb chemical compounds via van der Waals interaction [4]. The oxidation of graphene not only leads to surface defects but also decreases its hydrophobic features. The oxygenated functional groups of graphene oxide (GO) can be beneficial for the functionalisation of the GO surface via covalent bonds as well as noncovalent interactions [5]. The biomedical application of reduced GO (rGO) and GO is under investigation because of the possibility for surface modification and other biological features of graphene, the likelihood of crossing cell membranes [6], high affinity to the cell body [7], interaction with genetic materials [8] or accumulation at the administration site [9]. The construction of graphene-based complexes is constantly studied, notably in the delivery of drugs, bioactive molecules and genetic materials. Furthermore, graphene-based complexes have been estimated as a nonviral and effective gene delivery system for cancer therapy [10]. GO and rGO can bind nucleic acids at a high capacity [11]. The carbon hexagonal structure of GO can be loaded with both single-stranded DNA and RNA based on π–π stacking and hydrophobic interactions between the GO structure and the aromatic rings of the nucleic acid structure [12]. Additionally, published data have shown that GO had a protective effect on single-stranded DNA, protected from the enzymatic activity of DNAse I, which make GO nanoplatforms nucleic acid–protective [13]. GO and rGO have been used as delivery platforms for different types of genetic materials, including plasmid DNA [14], small interfering RNA [15,16], single-stranded RNA [17,18] and microRNA (miRNA) [19,20]. However, most of these studies used additional chemical functionalisation of GO or rGO complexes. There is limited data on the functionalisation of GO and rGO complexes only by the self-organisation of miRNA.

A miRNA is a small (18–22 nucleotides long), single-stranded, evolutionary conserved, noncoding functional sequence of RNA. A miRNA can post-transcriptionally regulate gene expression by complementarily binding to the 3’ region (UTR) of target mRNA. The function of miRNAs is the negative regulation of the expression of mRNA through the base-pairing of miRNAs with complementary-target mRNA [21]. The level of complementarity between miRNAs and the target sequence of mRNA determines the silencing mechanism, which is the mediated target mRNA cleavage, inhibition of translations into proteins or destabilisation of the mRNA through the shortening of its poly(A) tail [22]. miRNAs are involved in over 60% of all protein expression in humans, and a single miRNA sequence can modulate different pathways by regulating the expression of many target RNAs [23]. Moreover, the expression of miRNAs is highly dysregulated in cancer [21] and other types of cellular diseases, especially in neurodegenerative disorders [24].

Cancer development involves the dysregulation of the gene expression profile associated with cell proliferation, differentiation or cell death. miRNA expression in cancer has been intensively studied because of its vital role in regulating the metabolic and cellular pathways of cancer cells [25]. The idea to use miRNAs against cancer is based on their ability to target different genes involved in different cancer pathways. miRNAs can act as mimic structures to an increased level of mRNA of target genes or can react as an antisense miRNA and cause gene silencing in cancer cells [26]. The mimic structure of miRNAs should stimulate the endogenous structure and behaviour of miRNAs; thus, double-stranded miRNA mimics are preferred and can be incorporated into the RNA-induced silencing complex (RISC) [27]. Mimic miRNAs, after incorporation into the RISC, cause translational inhibition with target mRNA degradation or sequestration in P-bodies at the cytoplasm [28].

Glioblastoma (GBM) is the most common primary brain tumour with a poor survival rate of 14–15 months after diagnosis [29]. Despite extensive research, the efficiency of GBM therapy is low, and there is a high need for treatment improvements [30]. Gene therapy for GBM based on antisense miRNA can be a resolution for tumour regression and improvement of patients’ survival rate. However, the limitation of the usage of viral vectors for miRNA delivery involves immunogenicity, and it has to be eliminated [31]. Several studies point out the side effects of viral vectors in gene therapy, including immunoreactivity with high stimulation of the immune system, degradation of transfected tissue, mutagenic features, insufficient targeting of selected cells and limitations in gene packaging size capacity [32]. The immunogenicity of gene therapy can be limited by nonviral vectors including usage of nanosized materials [33].

In GBM cells, there are several downregulated sequences of miRNA [34], including miRNA-124 and miRNA-137 [23], acting as tumour suppressors [5]. Moreover, at the GBM stem cell (GCS) population, the synergic antitumour activity of miRNA-124 and miRNA-137 was observed, which causes the decreasing level of GSC proliferation and a higher level of sensitisation to chemotherapy and radiation [35]. The miRNA-124 and -137 target genes are associated with proliferation and can arrest the cell cycle at the G0/G1 stage of GBM [36]. Antisense miRNAs are single-stranded RNA oligonucleotides that complement the target miRNAs. The binding between antisense miRNAs and target miRNA sequences inhibits RISC activity and causes downstream miRNA expression [37]. miRNAs that are upregulated in cancer, such as miRNA-21 [38], miRNA-221 and miRNA-222 [39], are associated with GBM. The overexpression of miRNA-21 is observed in over 70% of patients diagnosed with GBM, and it is considered as mostly upregulated sequences of miRNAs in gliomas. In GBM, the decreased level of miRNA-21 leads to the activation of apoptotic cell death and reduces the malignancy and invasiveness of cancer [40]. Gene therapy with antisense miRNA-21 targets several tumour suppressor genes [41,42,43]. The cluster of oncomirs-221/222 targets over 70 genes associated with survival, proliferation, progression and invasiveness of GBM cells [44]. In the U251 GBM cell line, the protein expression of phosphatase and tensin homolog deleted on chromosome ten (PTEN), as well as GBM cell proliferation, decreased after the delivery of antisense miRNA-221 and miRNA-222 [43]. Moreover, it was found that the upregulation of the miRNA-221/222 cluster increased tumourigenesis and the invasiveness of tumours [45].

The goal of this study was the effective delivery of mimic miRNA-124, miRNA-137, antisense miRNA-21, miRNA-221 and miRNA-222 into U87, U118, U87, U251 and T98 GBM and human foetal foreskin fibroblast (HFFF-2) cell lines by graphene-based complexes. The selected cell lines are genetically, morphologically, metabolically different but all of them were established non primary cell lines. The differences in cell lines sensitivity for graphene complexes introduction provides general information about its toxicology and effectiveness of anticancer activity. The HFFF-2 cell line was chosen for the investigation as a non-cancer cell line for observation of efficient miRNA delivery at normal cell line. The evaluation of the selected gene expression level after transfection vs. electroporation, associated with proliferation, apoptosis and cell survival, was the second goal. Here, we present data that offer considerable hope for utilising graphene-based complexes functionalised with miRNAs as future prospects in GBM therapy.

## 2. Results and Discussion

**Figure 1:**
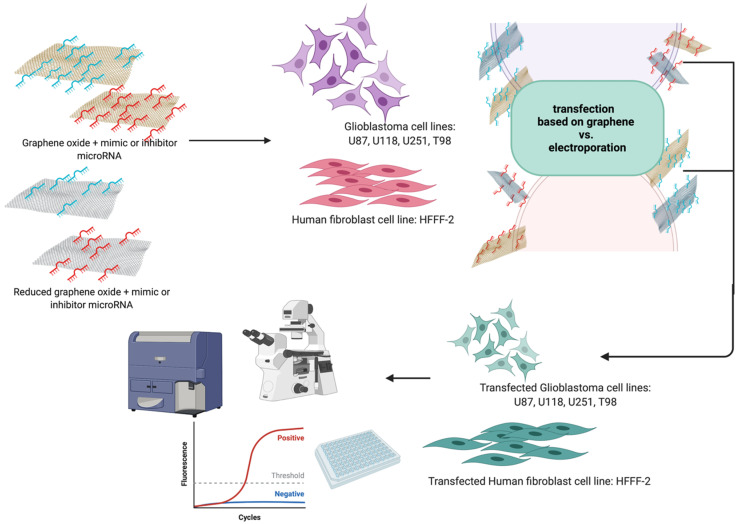
Graphical presentation of work.

The present results were obtained after the introduction of the hydrocolloids of GO, rGO, GO + miRNAs and rGO + miRNAs into the U87, U118, U251 and T98 GBM cell lines and the noncancer HFFF2 human fibroblast cell line. We hypothesised that graphene-based complexes increase the transfection efficiency of mimic miRNA and antisense miRNA delivery into GBM cancer cells (Figure 1). In this study, we observed the positive effect of in-situ GO and rGO functionalisation by selected sequences of miRNA.

### 2.1. Preparation and Characterisation of Graphene-Based Colloids

**Figure 2:**
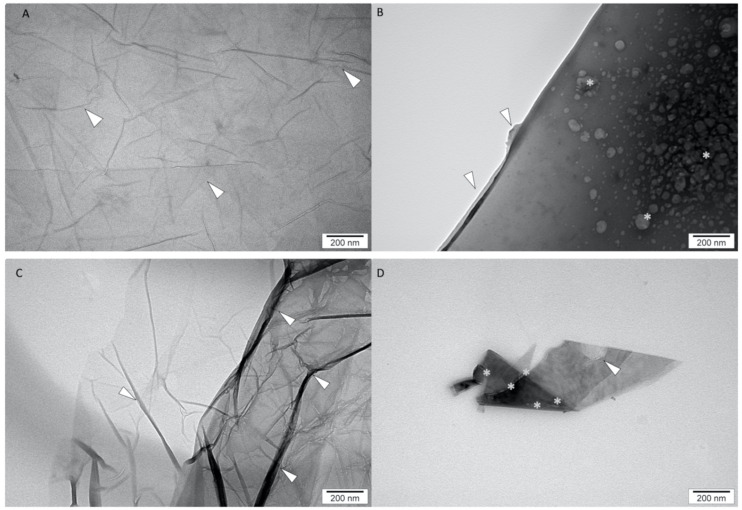
Transmission electron microscopy images of graphene oxide (**A**), reduced graphene oxide (**C**), graphene oxide functionalised with miRNA (**B**) and reduced graphene oxide functionalised with miRNA (**D**). Arrows: GO, rGO fakes corrugation, * miRNA, Scale bars: 200 nm.

The ultrastructure of GO, rGO, GO + miRNAs and rGO + miRNAs was inspected using scanning transmission electron microscopy. Figure 2 shows the representative transmission electron microscopies images.

The visualisation of the ultrastructure of GO (Figure 2A) showed multilayers of the GO sheet. Compared with the GO ultrastructure, rGO sheets showed a strong corrugation of flakes (Figure 2B). The morphology of rGO also showed that the sheets had an irregular shape and had the ability to agglomerate. Szczepaniak et al. [46] also observed the affinity to agglomerate GO and rGO sheets and different densities in images from transmission electron microscopy, which indicated a different number of layers in nanosheets. The in-situ functionalisation of GO and rGO nanosheets by miRNA sequences showed a high affinity of miRNA to the surface of the sheets (Figure 2B, D). The morphology of obtained GO + miRNA complexes showed a homogeneous distribution of miRNA oligonucleotides over the graphene surface (Figure 2B). The agglomeration of miRNAs was not detected (Figure 2B,D). The morphology of rGO + miRNAs showed a low dispersal of miRNAs. The similarity of the morphology ultrastructure of GO sheets after functionalisation was presented in our previous studies, where platinum nanoparticles were efficiently combined with the GO sheet surface via van der Waals forces [47]. The obtained results from dynamic light scattering (DLS) show that the average size of GO, rGO, GO + miRNA and rGO + miRNA was: 875.5 d. nm (PDI = 0.397), 1773.0 d. nm (PDI = 0.493), 805.0 d. nm (PDI = 0.305) and 2049.5 d. nm (PDI = 0.458). The DLS measures the translational diffusion constant of the particles inside using the Stokes-Einstein equation to deduce the particle radius. The Stokes-Einstein assumes that the measured particles are spherical. Consequently, the big sheet size of GO, rGO, GO + miRNA, and rGO + miRNA obtained by DLS could be falsely bigger than results obtained from TEM. TEM observation showed that the size of GO and rGO flakes was higher than 100 nm in diameter. However, the TEM images showed that the thickness of GO, rGO nanosheets, and complexes was extremely low (below 1 nm) (Figure 2). The graphene does not have rotational symmetry, so DLS measured could give falsely bigger nanosheets size results. The polydispersity index (PDI) at all investigated samples was higher than 0.300, indicating a wide particle size distribution. The supramolecular features of the graphene surface and covalent bonding were utilised to obtain graphene nanoplatforms functionalised by miRNA-15a [48].

**Figure 3:**
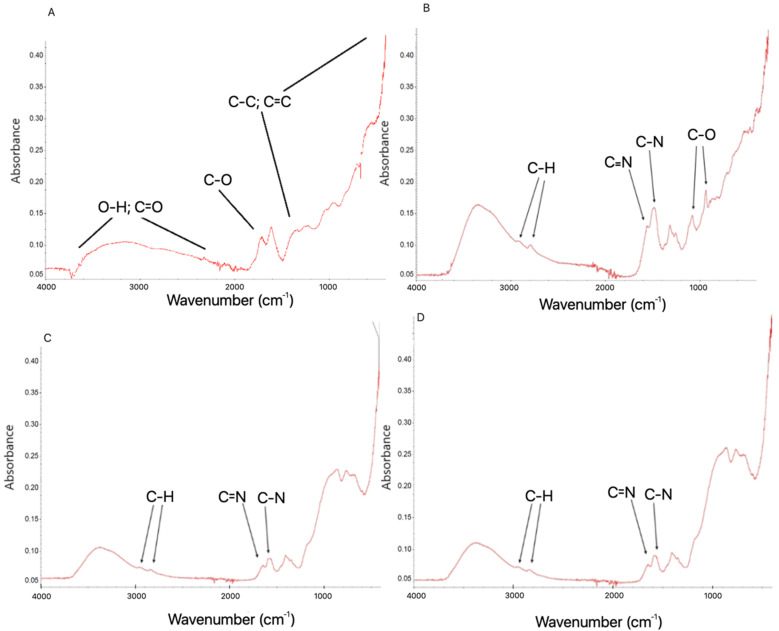
Fourier transform infrared spectrum of graphene oxide (**A**), reduced graphene oxide (**C**) and complexes of graphene oxide + miRNA (**B**) and reduced graphene oxide + miRNA (**D**). Abbreviations: GO: graphene oxide, rGO: reduced graphene oxide, GO + miRNA: complexes of graphene oxide functionalised with miRNA, GO + miRNA: complexes of reduced graphene oxide functionalised with miRNA.

The FTIR spectra (Figure 3) after functionalisation of GO sheets by miRNAs (Figure 3B) showed signals at wavenumbers of 1700 cm^−1^, 1650 cm^−1^ and 1200 cm^−1^, which are associated with C=O, C=C and C-O bonds of GO, respectively. The results also show a high signal starting from around 1050 cm^−1^, which corresponds to C-C wagging. Finally, the broad signal of around 3400 cm^−1^ is a result of the O-H stretching of hydroxyl groups in GO and confirmed the presence of the -OH group in the analysed sample. The two characteristic bands were observed to be caused by C-H stretching at region 2850–2950 cm^−1^. The rGO spectra after the functionalisation by miRNAs (Figure 3D) showed signals at region 2840–2950 cm^−1^, corresponding to strong C-H stretching. Two bands located at 1588 cm^−1^ and 140 cm^−1^ can be assigned to C=C and C-H, respectively. The results of the FTIR spectra from our study show that the functionalisation of GO surfaces by miRNA was the result of noncovalent bonding and did not change the simple chemical composition of the surface of GO and rGO sheets.

The obtained results from FTIR analysis were confirmed by TEM observation of the ultrastructure, where miRNA was located only at GO/rGO sheets surface. In the observation of ultrastructure of GO/rGO + miRNA, no free miRNA particles were found outside the area of the graphene sheets. This observation with FTIR spectrum analysis confirms the efficiency of functionalization GO/rGO with miRNA. The measurements of entrapment efficiency (EE) showed a high level of entrapment for GO (87.80% ± 2.25%) and rGO (95.45% ± 0.50%). Graphene sheets have a high capacity to entrap nucleotides to form compact graphene-based complexes [49]. The unique physiochemical features of graphene oxide and reduced graphene oxide increased the functionalization possibility and its EE. The EE provided supporting data for the FTIR and TEM results. The characteristics band in the FTIR spectra of the investigated samples were similar to the spectra of GO and rGO obtained by Kutwin et al. [47], Szczepaniak et al. [46]. The ζ-potential for GO and rGO was −21.7 mV and −9.29 mV, respectively. GO was the most stable colloid of the nanoparticles. The functionalisation of GO or rGO sheets by miRNA did not increase the stability of GO and rGO colloids. The ζ-potential for GO + miRNAs and rGO + miRNAs was −12.1 mV and −12.6 mV, respectively.

### 2.2. Cell Cultures and Treatments

**Figure 4:**
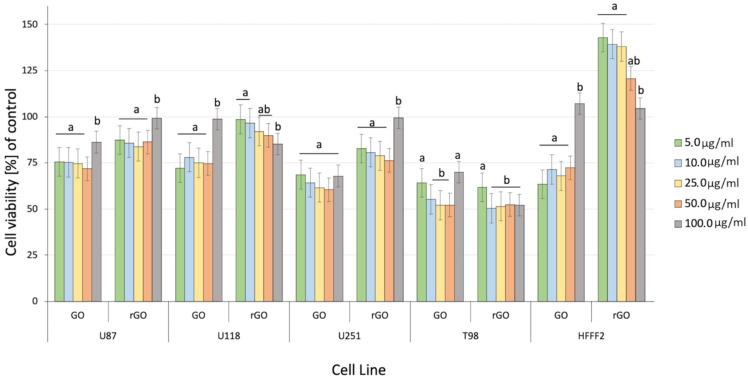
Effect of graphene oxide (GO) and reduced graphene oxide (rGO) on U87, U118, U251 and T98 GBM cell lines and HFFF-2 human fibroblast viability. Bars represent the means with standard deviation (SD) (*n* = 3). Notes: Different lowercase letters (a and b) within columns indicate significant differences between the concentrations (*p* < 0.05). Abbreviations: GO: graphene oxide and rGO: reduced graphene oxide.

The assessment of cell viability after GO and rGO treatment showed decreased viability at all GBM cell lines (Figure 4). The GO treatment significantly affects cell viability at all investigated cell lines and concentrations (*p*-value = 0.00052). The comparison of the GBM effect of treatment between GO and rGO at 5.00 µg/mL showed the highest and most important difference between the treated and nontreated groups. The differences in the viability of cancer cells were significant compared to the nontreated control group, especially at a dose of 50 µg/mL of GO. The differences between cell line and type of graphene were significant.

**Figure 5:**
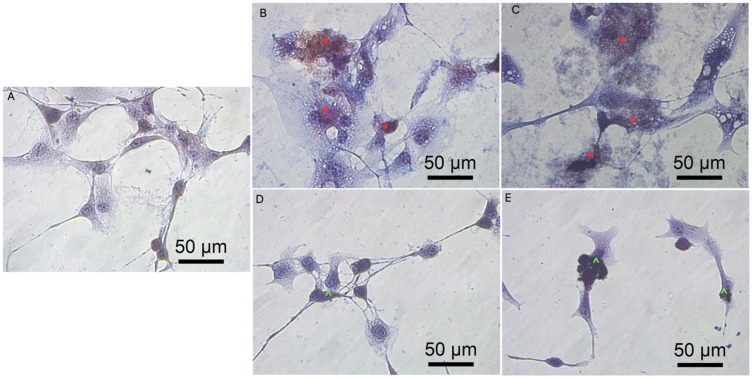
Morphology of U87 glioblastoma cells. (**A**) untreated cells (control group), cells treated with graphene oxide at concentration: 5μg/mL (**B**), 100μg/mL (**C**), and reduced graphene oxide at concentration: 5 μg/mL (**D**) and 100 μg/mL (**E**). Red * GO at cell membrane. Green ^ rGO at cell membrane. Light optical microscopy. Scale bars: 50 μm. (**A**–**E**). Abbreviations: GO: graphene oxide, rGO: reduced graphene oxide.

**Figure 6:**
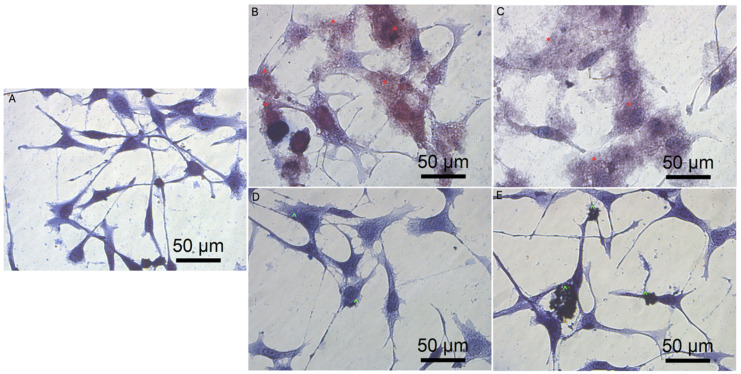
Morphology of U118 glioblastoma cells. (**A**) untreated cells (control group), cells treated with graphene oxide at concentration: 5μg/mL (**B**), 100μg/mL (**C**), and reduced graphene oxide at concentration: 5 μg/mL (**D**) and 100 μg/mL (**E**). Red * GO at cell membrane. Green ^ rGO at cell membrane. Light optical microscopy. Scale bars: 50 μm. (**A**–**E**). Abbreviations: GO: graphene oxide, rGO: reduced graphene oxide.

**Figure 7:**
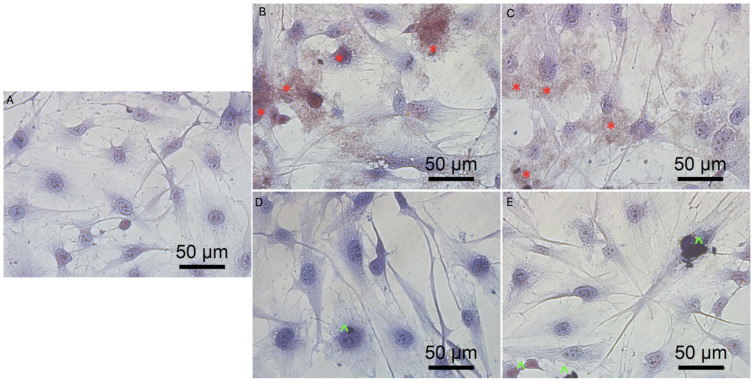
Morphology of U251 glioblastoma cells. (**A**) untreated cells (control group), cells treated with graphene oxide at concentration: 5 μg/mL (**B**), 100 μg/mL (**C**), and reduced graphene oxide at concentration: 5 μg/mL (**D**) and 100 μg/mL (**E**). Red * GO at cell membrane. Green ^ rGO at cell membrane. Light optical microscopy. Scale bars: 50 μm. (**A**–**E**). Abbreviations: GO: graphene oxide, rGO: reduced graphene oxide.

**Figure 8:**
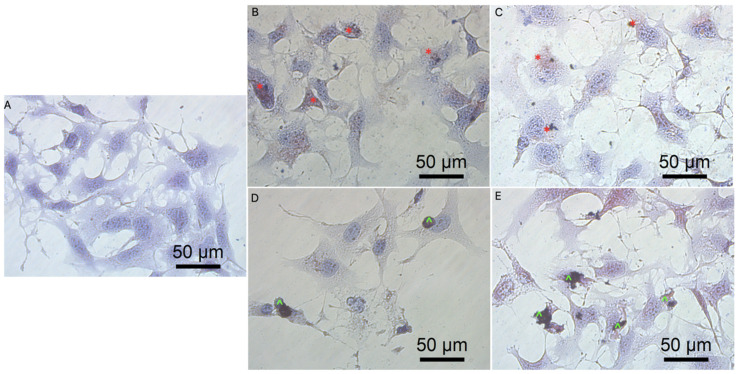
Morphology of T98 glioblastoma cells. (**A**) untreated cells (control group), cells treated with graphene oxide at concentration: 5 μg/mL (**B**), 100 μg/mL (**C**), and reduced graphene oxide at concentration: 5 μg/mL (**D**) and 100 μg/mL (**E**). Red * GO at cell membrane. Green ^ rGO at cell membrane. Light optical microscopy. Scale bars: 50 μm. (**A**–**E**). Abbreviations: GO: graphene oxide, rGO: reduced graphene oxide.

**Figure 9:**
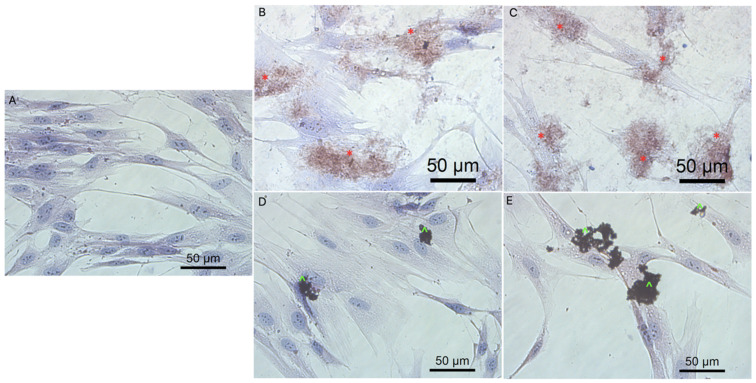
Morphology of HFFF-2 fibroblast cell line. (**A**) untreated cells (control group), cells treated with graphene oxide at concentration: 5 μg/mL (**B**), 100 μg/mL (**C**), and reduced graphene oxide at concentration: 5 μg/mL (**D**) and 100 μg/mL (**E**). Red * GO at cell membrane. Green ^ rGO at cell membrane. Light optical microscopy. Scale bars: 50 μm. (**A**–**E**). Abbreviations: GO: graphene oxide, rGO: reduced graphene oxide.

Previous data also indicated that GO had an anticancer effect against several cancer cell lines [7,46]. The obtained results show that rGO decreased GBM cell viability at a minor level, which can be associated with poor solubility, and increased the agglomeration of rGO [50]. The noncancer HFFF-2 cell line was resistant to the rGO treatment and showed increased viability at all inspected concentrations (Figure 4). The GO treatment stimulated the growth of the cell only at the highest concentration (100 µg/mL). The higher concentration of GO and rGO caused cytotoxicity limitation at U87, U118, U251 GBM cell line. A total of 100 µg/mL of GO increased the vitality of T98 GBM cell line and HFFF-2 human fibroblast cell line. GO at the highest concentration tended towards agglomeration and increased its biocompatibility in in vivo studies [9]. Similar findings were published by Liao [51], where rGO was less harmful than GO against human fibroblast. Moreover, the morphology assessment of GO and rGO treatments of U87 (Figure 5; Figure S1, see Supplementary Materials), U118 (Figure 6; Figure S2), U251 (Figure 7; Figure S3) and T98 (Figure 8; Figure S4) GBM cell lines and the HFFF-2 (Figure 9; Figure S5) cell line showed that GO and rGO had a strong affinity to the cell membrane and decreased the density of GBM cells at higher concentrations of GO and rGO. Lower concentrations of GO and rGO decreased cell size and shortened cell tentacles. Agglomeration of rGO was also detected during the cell morphology observation, indicating that GO colloids were more stable and had larger and unblocked active agglomeration surfaces. Based on the obtained results for cell viability and morphology, GO at a concentration of 100 µg/mL was chosen for complex synthesis. A similar observation was presented by Campbell et al. [52], where GO was identified as an effective nontoxic delivery platform with high internalisation efficiency in different types of cancer cells. The partial resistance of the U87 cell line to GO treatment can be caused by the physiochemical features and surface modification of GO. The results confirm previously published data, in which GO treatment was less harmful to U87 glioma cells than rGO [53]. Published data also indicated that the physicochemical property of GO increased the efficiency of siRNA gene delivery into the cancer cells [54] with limited cytotoxic effect [55].

**Figure 10:**
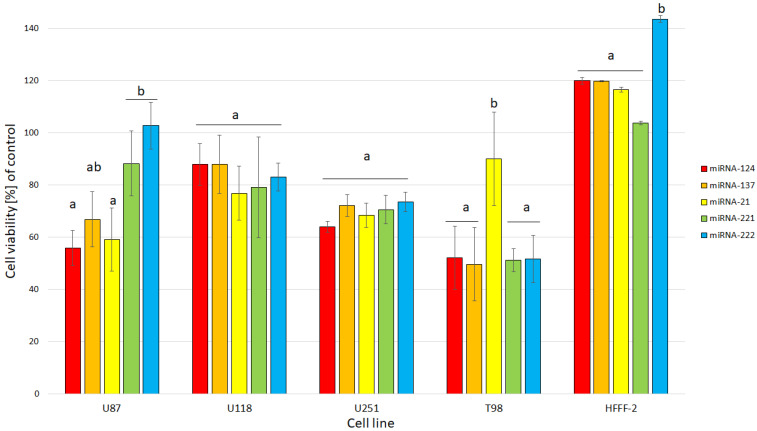
Effect of miRNA-124, miRNA-137 and antisense miRNA-21, -221 and -222 on U87, U118, U251 and T98 GBM cell lines and HFFF-2 human fibroblast viability. Bars represent means with standard deviation (SD) (*n* = 3). Notes: Different lowercase letters (a, b) within columns indicate significant differences between the miRNA (*p* < 0.05).

**Figure 11:**
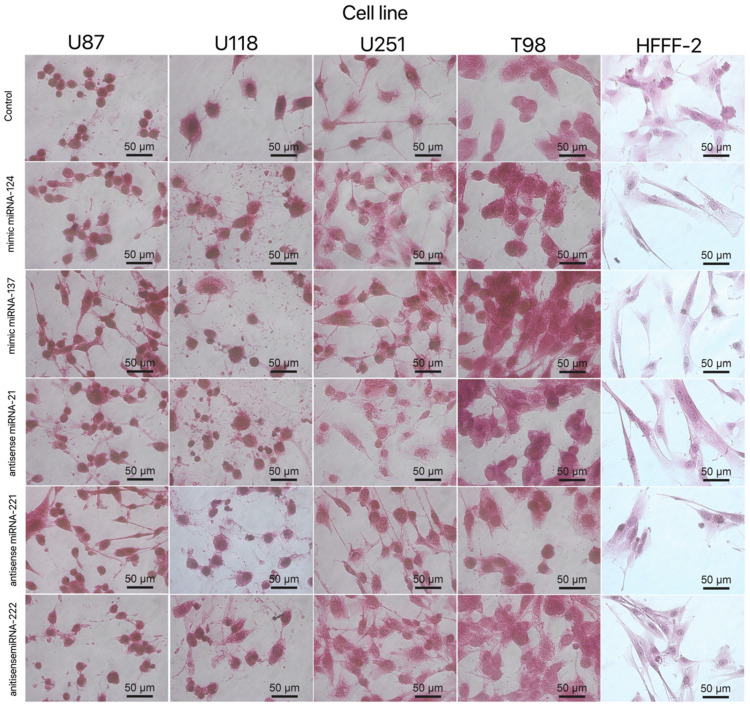
Morphology of U87, U118, U251 and T98 glioblastoma and HFFF-2 cells treated with mimic miRNA-124, mimic miRNA-137, antisense miRNA-21, antisense miRNA-221 and antisense miRNA-222. Scale bars: 50 μm.

The obtained results for the viability of GBM and the noncancer cell line after electroporation show that mimic and antisense sequences of selected miRNA decreased cell viability (Figure 10). The mimic sequences miRNA-124 and miRNA-137 significantly reduced cell viability in U87, U251 and T98 GBM cell lines. The published data indicated the antitumoural activity of miRNA-124 and -137 in GBM cancer therapy [35]. Moreover, the morphology of the GBM cell line and HFFF-2 cells (Figure 11) showed that all investigated sequences of miRNA affected cell morphology observed through a reduction in the number and size of tentacles and sharpened nuclei, limited cell-to-cell interaction and loss of typical cell arrangement. Additionally, GBM cell line transfection by miRNA-21, miRNA-221 and miRNA-222 decreased the viability of GBM cells.

**Figure 12:**
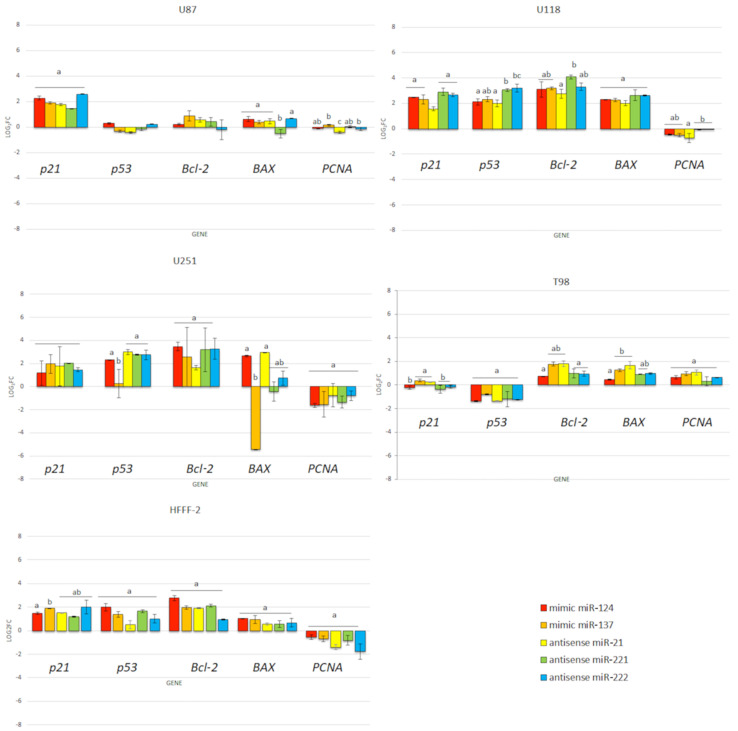
Expression of genes p21, p53, Bcl-2, BAX and PCNA at the mRNA level in U87, U118, U251 and T98 glioblastoma and HFFF-2 fibroblast cells 24 h post transfection by electroporation using RT-PCR. Bars represent means with standard deviation (SD) (*n* = 3). Relative expression was calculated using the gapdh gene and the control group (0). The results are presented as log_2_FC values. Values above/below 0 indicate upregulation/downregulation of gene expression. FC fold change. Notes: Different lowercase letters (a, b) within columns indicate significant differences between the miRNA (*p* < 0.05).

The mimic miRNA-124 and -137 transfections of GBM cells had the smallest impact on the reduction in disorganised arrangement in cell culture (Figure 11). The antisense miRNA-21 at all investigated cell lines reduced cell density, the number of cell tentacles and the size of the cell body. Zhang et al. [40] also indicated that the downregulated level of miRNA-21 may reduce the malignancy and invasiveness of GBM. Moreover, 24 h post transfection, the morphology of GBM cells did not present characteristic features of cancer cells, including large variability and sharpened nuclei, many dividing cells or variation in the size and shape of the cell body. The antisense miRNA-221 and -222 caused a similar cell aberration in the cellular structure to the antisense miRNA activity against the GBM cell lines. To confirm the essential role of the selected sequence of miRNA on the activation of the signalling pathway involved in apoptosis and proliferation, the p21, p53, Bcl-2, Bcl-2-associated X protein (BAX) and proliferating cell nuclear antigen (PCNA) gene expression level were investigated (Figure 12). Transfection by electroporation did not cause significant changes at the gene expression level in the U87 GBM cell line. The proapoptotic BAX gene expression level was upregulated by the delivery of mimic miRNA-123, -137 and antisense miRNA-21 and -222 at the U87 GBM cell line. The most sensitive for miRNA transfection was the U118 GBM cell line, with the highest upregulation of the BAX gene and a significant silencing of the PCNA gene expression level. The anticancer activity of antisense miRNA-21 was also observed in the inhibition of the proliferation of cancer cells and the reduced viability of the U118 GBM cell line. The observation was similar in the U251 GBM cell line. In the noncancer HFFF-2 cell line, the activation of apoptosis was observed by the upregulation of the BAX expression (Figure 12), deformation of cell morphology (Figure 11) and reduction in cell viability (Figure 10).

**Figure 13:**
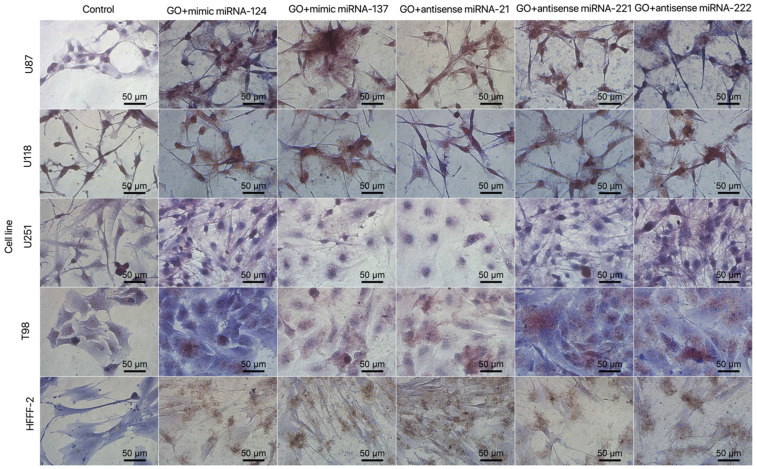
Morphology of U87, U118, U251 and T98 glioblastoma and HFFF-2 cells treated with GO + mimic miRNA-124, GO + mimic miRNA-137, GO + antisense miRNA-21, GO + antisense miRNA-221 and GO + antisense miRNA-222. Abbreviations: GO: graphene oxide. Scale bars: 50 μm.

**Figure 14:**
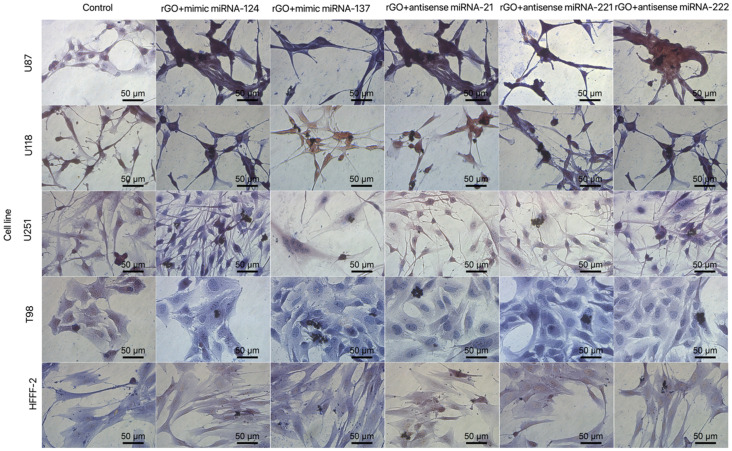
Morphology of U87, U118, U251 and T98 glioblastoma and HFFF-2 cells treated with rGO + mimic miRNA-124, rGO + mimic miRNA-137, rGO + antisense miRNA-21, rGO + antisense miRNA-221 and rGO + antisense miRNA-222. Light optical microscopy. Abbreviations: rGO: reduced graphene oxide. Scale bars: 50 μm.

**Figure 15:**
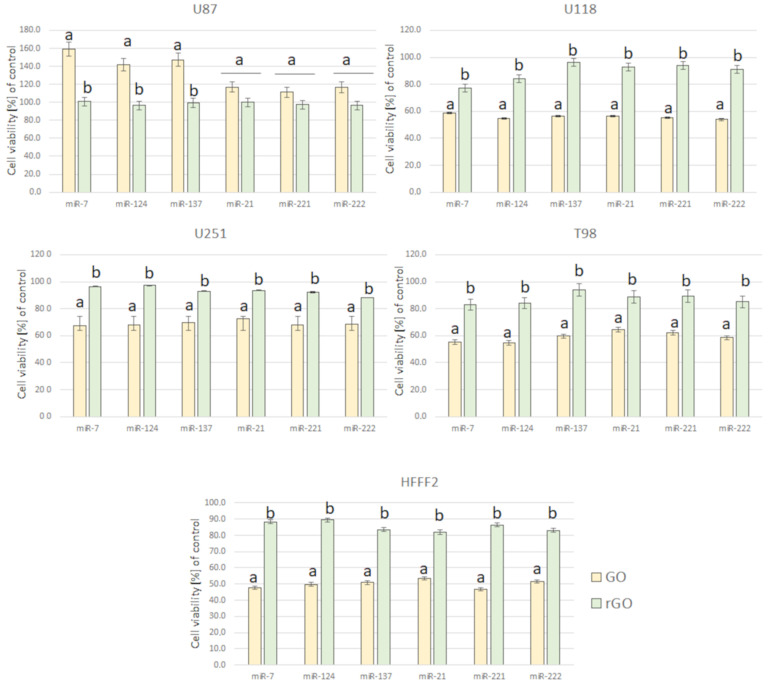
Effect of complexes of GO + miRNA and rGO + miRNA on U87, U118, U251 and T98 GBM cell lines and HFFF-2 human fibroblast viability. Notes: Different lowercase letters (a, b) within columns indicate significant differences between the miRNAs (*p* < 0.05). Abbreviations: GO: graphene oxide, rGO: reduced graphene oxide.

High proliferation level and resistance to apoptosis are fundamental parts of carcinogenesis. One of the critical pathways involved in cancer development is p53, which is responsible for detecting DNA damage and indirectly activates apoptosis signalling pathways. p53 affects tumour suppression by activating selected genes involved in cell cycle arrest [56], growth arrest, apoptosis, DNA repair and senescence [57,58]. Moreover, the p53 pathway is one of the most frequently deregulated genes in GBM and leads to high proliferation and progression and determines the high invasive growth of GBM tissue [59]. The p53 pathway in cancer is also indirectly regulated by noncoding transcripts including miRNA-124 [60], miRNA-137 [61], miRNA-21 [62] and miRNA-221 and -222 [63]. The miRNA transfection of GBM cells affects not only cell morphology and viability but also indicates that the selected sequences of miRNA affect the expression level of p53 and cause its upregulation in the U118 and U251 cell lines. Moreover, the comparison of cell morphology and viability between GO_100 µg/mL_ + miRNA and rGO_100 µg/mL_ + miRNA nano transfection showed that both types of graphene interacted with the GBM cell body and caused serious cell deformation (Figure 13 and Figure 14), leading to decreased cell viability (Figure 15).

**Figure 16:**
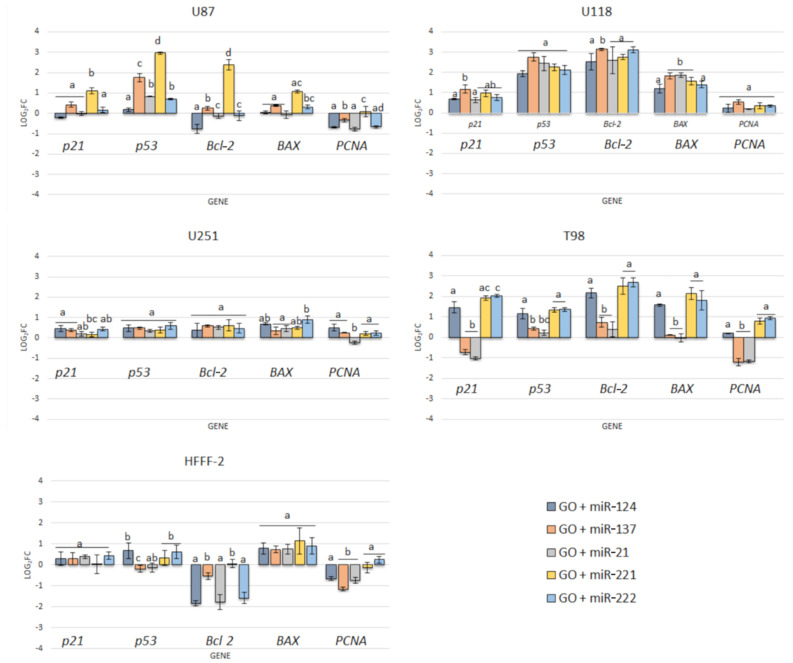
Expression of genes p21, p53, Bcl-2, BAX and PCNA at the mRNA level in U87, U118, U251 and T98 glioblastoma and HFFF-2 fibroblast cells after 24 h graphene oxide + miRNA nano transfection using RT-PCR. Bars represent means with standard deviation (SD) (*n* = 3). Relative expression was calculated using the gapdh gene and the control group (0). The results are presented as log_2_FC values. Values above/below 0 indicate upregulation/downregulation of gene expression. FC fold change. Notes: Different lowercase letters (a, b) within columns indicate significant differences between the miRNAs (*p* < 0.05).

In our studies, the GO_100 µg/mL_ + miRNAs showed the most efficient anticancer effect of treatment at the gene expression level compared to electroporation. The partial resistance of the U87 cell line for the GO + miRNA treatment can be caused by the physiochemical features and surface modification of GO. The results confirm previously published data, where GO treatment was less harmful to U87 glioma cells than rGO [53]. Moreover, the synergy of GO and miRNA activity against the viability of GBM cancer cells showed that GO-based complexes are promising as future anticancer therapy. Recently, the standard therapy for GBM has been surgery resection and chemotherapy with temozolomide (TMZ) and radiation. The premedication with TMZ increased the efficiency of radiation. TMZ is a monofunctional DNA alkylating agent of the imidazotetrazine class. The mechanism of action is related to the methylation of DNA and the active form of TMZ(5-(3-methyl-triazen-1-yl)imidazole-4-carboxamide (MTIC)) preferentially methylates DNA at N7 positions of guanine in guanine rich regions of GBM cells. However, the structure of TMZ could be protected from degradation in the blood stream by a graphene-based carrier and increase its local anticancer activity [64]. The results of the effect of GO + miRNA on the selected gene expression level (Figure 16) show that GO + miRNA complexes were more effective than electroporation of GBM with selected sequences of miRNA. The downregulation of miRNA-124-mediated malignant progression of GBM is partly involved in the overexpression of inhibitory members of the apoptosis-stimulating protein of p53 [56]. Moreover, a reduction in the expression level of miRNA-124 and -137 showed the synergism of action by interaction with target genes involved in the proliferation and invasion of cancer cells [57].

Based on the obtained results of these studies, the most stable anticancer features showed that GO + antisense miRNA-21 was observed to decrease cancer cell viability (Figure 10) and cell morphology deformation (Figure 11) and increase the expression level of proapoptotic genes (Figure 12). The selection of antisense miRNA-21 was done for the next step of the investigation, where GO + antisense miRNA-21 was investigated regarding the effectivity of transfection and activation of programmed cell death by apoptosis.

miRNA-21 was described as an antiapoptotic factor in GBM with a 5–100-fold change in expression compared to non-neoplastic tissue [42]. The sequence of the antisense miRNA-21 was identified as a factor for the knockout of miRNA-21 gene expression and caused decreased viability of the U251 and LN229 GBM cell lines [40]. miRNA-21 was the first miRNA sequence associated with the deregulation of apoptosis in GBM [65]. The silencing of miRNA-21 could decrease proliferation and GBM tumour growth. The direct mechanism of proliferation inhibition was based on the downregulation of miRNA-21 expression and activation of caspases and consequently increases apoptotic cell death in GBM [42].

In our studies, the GO + antisense miRNA-21 showed a more efficient anticancer effect of treatment than transfection by electroporation and upregulated the expression level of proapoptotic genes (BAX) and downregulated the expression of PCNA and Bcl-2 (Figure 16).

**Figure 17:**
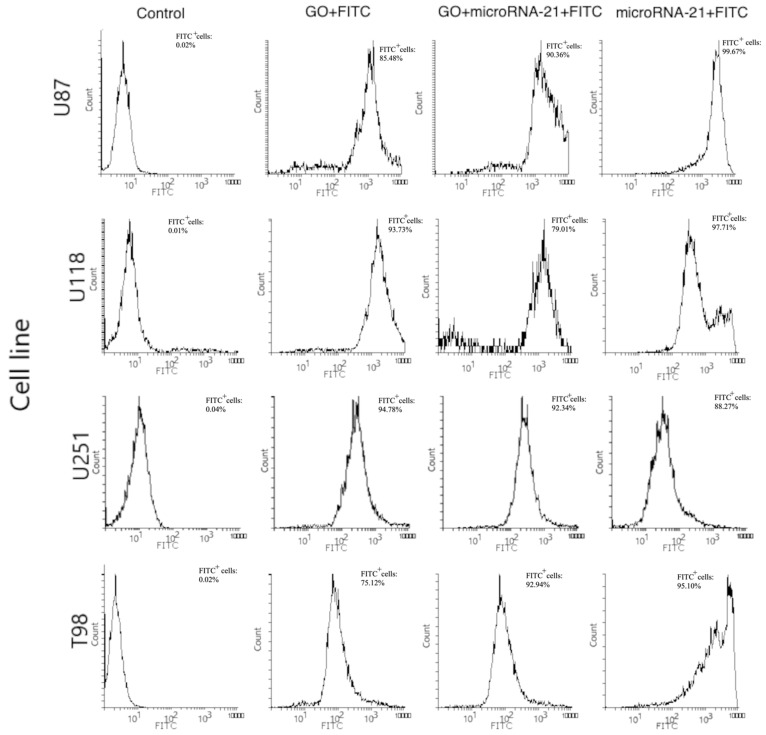
Flow cytometric analysis of unstained and FITC-stained cells of U87, U118, U251, T98. Forward scatter (FSC) vs. side scatter (SSC) two-dimensional (2D) dot plots of unstained cell population (Control), FITC-labelled GO cell population (GO + FITC), FITC-labelled GO + antisense miRNA-21-stained cell population (GO + miRNA-21 + FITC), FITC labelled antisense miRNA-21-stained cell population (miRNA-21 + FITC). Abbreviations: FITC^+^—FITC positive cells.

In our study, transfection of GO + miRNA-21 caused the upregulation of p21, p53 and BAX genes at all investigated GBM cell lines. These findings indicated that apoptosis can be activated because of the silencing of the miRNA-21 expression. However, the expression level of the bcl-2 gene, which is a strong inhibitor of apoptosis, at the U118 GBM significantly increased after transfection with all investigated sequences of miRNA. At the U118 GBM also, the expression level of the BAX gene increased after transfection with antisense miRNA-21, and the BAX/bcl-2 ratio was 0.719. Kiprian et al. [66] indicated that the BAX/bcl-2 ratio, as a proapoptotic indicator, was more reliable than the expression level of individual genes. GO + antisense miRNA- 21 also downregulated the expression of PCNA at the U87, U251 and T98 GBM cell lines. PCNA is a molecular marker of the proliferative phase of the cell cycle and plays an important role in the metabolism of nucleic acid. It is essential for DNA replication and is also involved in DNA excision repair, chromatin assembly, cell cycle control and RNA transcription [67]. The interaction between PCNA and p21 was also evaluated. p21 interacts with PCNA and caused the inhibition of DNA synthesis by displacing DNA polymerases and other proteins associated with DNA replication [67]. In our study, p21 expression level was upregulated by GO + antisense miRNA-21 in the U87, U118 and U251 GBM cell lines, as well as in the HFFF-2 noncancer cell line. Moreover, the expression level of p21 corresponded with the increased expression level of p53. p53 is responsible for inhibiting the neoplastic phenotype. If DNA damage is detected, it can arrest the phase G1 cell cycle and activate proteins involved in DNA repair. The upregulation of p53 was involved in triggering cell apoptosis by activating the BAX gene [68]. The delivery of miRNA-221 and -222 by GO through transfection leads to the upregulation of p21, p53 and BAX genes at all investigated GBM cell lines. However, antisense miRNA-221 and -222 did not decrease the proliferation at the investigated GBM cell lines except U87. The results also show that at HFFF-2, which was the noncancer control group, GO + antisense miRNAs-221 and -222 did not cause the increased expression of p21 and p53. A similar observation of decreased cell viability was presented after the transfection of antisense miRNA-221 and antisense miRNA-222 into the U251 GBM cell lines [40]. Based on RT-PCR analysis results, the activation of apoptosis at the protein level and transfection effectivity evaluation was done after GO + antisense miRNA-21 treatment of GBM cell lines was confirmed as the most promising anticancer complex. To confirm the effectivity of transfection of GO sheets, GO + antisense miRNA-21 and antisense miRNA-21 sequences were conjugated with fluorescein isothiocyanate, and the effect was observed by flow cytometry. The results show that the GO + antisense miRNA-21 sequence had similar transfection effectivity to electroporation (Figure 16). The level of fluorescein isothiocyanate–positive (FITC) cells after transfection by GO + miRNA was not lower compared to the obtained electroporation results (Figure 17). The histograms revealed that the cells, after transfection by electroporation or transfection, were labelled by FITC. These findings confirm that transfection by GO conjugated with selected miRNA sequences can be an efficient complex for gene sequence delivery.

**Figure 18:**
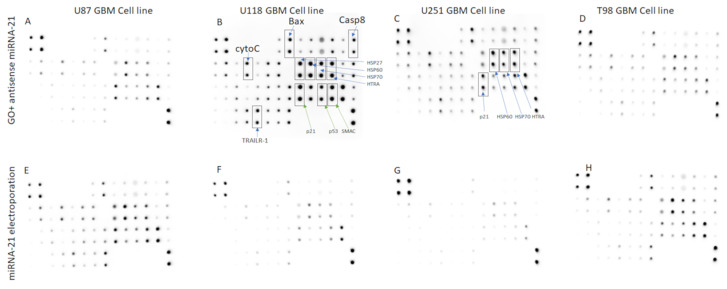
Antibody array analysis of proapoptopic protein synthesis in U87 (**A**,**E**), U118 (**B**,**F**), U251 (**C**,**G**) and T98 (**D**,**H**) glioma cells treated with complexes of graphene oxide functionalised with antisense miRNA-21 (**A**–**D**) and miRNA-21 by electroporation (**E**–**H**). GO + antisense miRNA-21 treatment results in the upregulation synthesis of Bcl-2-associated X protein (BAX), caspase-8, heat shock protein 27 (HSP27), heat shock protein 60 (HSP60), TNF-related apoptosis-inducing ligand (TRAILR-2), second mitochondria-derived activator of caspase (Smac) and cytochrome c in U118 (**B**) and U251 (**C**).

The results of the apoptosis assessment show that the U118 GBM cell line was the most sensitive for GO + antisense miRNA-21 treatment, and the activation of apoptosis was detected by the observation of the increasing expression level of selected proteins. (Figure 18).

**Figure 19:**
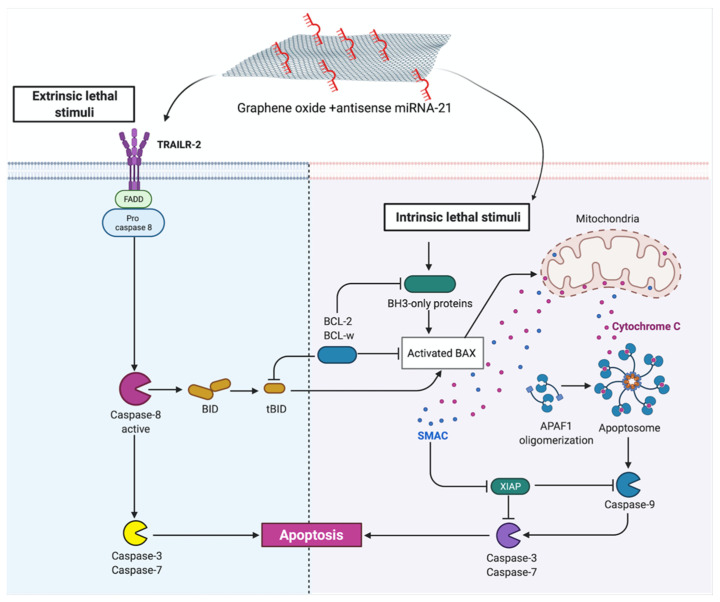
Protein interaction after graphene + antisense miRNA-21 complexes delivery into glioblastoma cells.

The expression level of proapoptotic protein such as a BAX, heat shock protein 60 (HSP60), caspase-8, TNF-related apoptosis-inducing ligand (TRAILR-2), second mitochondria-derived activator of caspase (Smac) and cytochrome c was increased after GO + antisense miRNA-21 treatment at the U118 GBM cell line. The results of apoptosis arrays indicate the bidirectional mechanism of apoptosis activation by GO + antisense miRNA-21. GO + antisense miRNA-21 treatment results in the upregulation synthesis of Bcl-2-associated X protein (BAX), caspase-8, heat shock protein 27 (HSP27), heat shock protein 60 (HSP60), TNF-related apoptosis-inducing ligand (TRAILR-2), second mitochondria-derived activator of caspase (Smac) and cytochrome c in U118 (Figure 18B) and U251 (Figure 18C). Apoptosis can be activated by intrinsic and extrinsic pathways in mammalian cells. The intrinsic pathway is controlled by Bcl2 protein family, whose members fall into three groups: proapoptotic BH3-only proteins, prosurvival Bcl-2-like proteins and the pore-forming BAX and Bak proteins [69]. BAX protein can damage the mitochondrial outer membrane to mediate cell death by apoptosis. Next, cytochrome c is released from the mitochondria, and once it is in the cytosol, it initiates the activation of caspases (aspartate-specific proteases) and cleavage of multiple cellular proteins. The second proapoptotic protein released from the intermembrane space of the mitochondria is Smac, which enhances caspase activation [69]. Releasing proapoptotic proteins such as cytochrome c and Smac from the intermembrane space of mitochondria indicated the role of transfection by GO + antisense miRNA-21 in an early step of apoptotic cell death. Meanwhile, the extrinsic apoptosis pathway is initiated through signal transduction mediated by members of the tumour necrosis factor (TNF) receptor superfamily [70]. The increased expression of TRAILR-2 and caspase-8 at the U118 GBM cell line indicated the activation of the extrinsic apoptosis pathway after GO + antisense miRNA-21 treatment. Moreover, the increased expression level of caspase-8, which is a member of the caspase family of cysteine proteases, indicated that transfection probably caused its activation by oligomerisation through autoproteolysis. The increased expression of p21 and p53 protein level stands in correspondence with gene expression level evaluated by qPCR analysis after GO + antisense miRNA-21 treatment of GBM cell lines. The activation of apoptosis by GO was also reported as a result of the interaction between several mechanisms, which was based on damage to the cell membrane and impaired mitochondrial activity [46]. Additionally, the inhibition of miRNA-21 expression leads to caspase activation, which is associated with apoptotic cell death in GBM cell lines [42]. In our study, the activation of apoptosis was bidirectional, which suggests the possible mechanism of action by the synergy of the physiochemical properties of GO and the biological activity of antisense miRNA-21 (Figure 19). The observation between complexes of GO vs. rGO and cell membrane are in agreement with published data, where GO presented a strong affinity to the cell membrane [7]. The developed GO membrane effectively delivered the miRNA observed by FITC + GO + miRNA intracellular activity at GBM cell lines. Moreover, GO + antisense miRNA-21 activated the programmed bidirectional cell death by the increased activity of BAX, CytoC and Smac protein, which indicated mitochondria dysfunction, and by the increased expression of caspase-8 and Trailr-2, which is associated with the extrinsic apoptosis pathway. These features of GO + antisense miRNA-21 can overcome the limitation of viral gene delivery [10] and is a promising new delivery complex for brain cancer therapy.

## 3. Materials and Methods

### 3.1. Preparation and Characterisation of Colloids

All measurements were performed in triplicate.

#### 3.1.1. Graphene Oxide and Reduced Graphene Oxide

GO colloid (GO) (purity 99.99%) was purchased from Nanopoz (Poznan, Poland) and rGO was purchased from Graphene Laboratories Inc. (Ronkonkoma, NY, USA) and dispersed in ultrapure water to prepare a 1.0 mg/mL solution.

After 30 min of sonification, the hydrocolloids of GO and rGO were diluted to different concentrations with 1× Dulbecco’s modified Eagle’s culture medium (Sigma-Aldrich, St Louis, MO, USA) immediately before exposure to cells.

#### 3.1.2. Complex of Graphene Oxide or Reduced Graphene Oxide with Antisense miRNAs

Suspensions of GO (100 μg/mL) and miRNA-124 (UAAGGCACGCGGUGAAUGCC; HMI0086; Sigma-Aldrich, Saint Louis, MI, USA), -137 (UUAUUGCUUAAGAAUACGCGUAG; HMI0207; Sigma-Aldrich, Saint Louis, MI, USA) and antisense miRNA-21 (UAGCUUAUCAGACUGAUGUUGA; HSTUD0399, Sigma-Aldrich, Saint Louis, MI, USA), -221 (ACCUGGCAUACAAUGUAGAUUU; HSTUD0397, Sigma-Aldrich, Saint Louis, MI, USA) or -222 (CUCAGUAGCCAGUGUAGAUCCU; HSTUD0371, Sigma-Aldrich, Saint Louis, MI, USA) at concentration 5 pmol/mL were prepared in ultrapure water and used without additional purification and filtration. Ultrasonic coating of GO or rGO nanosheets for 30 min took place in a 50 mL glass flask.

#### 3.1.3. Transmission Electron Microscopy

The shape and size of the GO, rGO, GO antisense miRNAs and rGO antisense miRNAs were inspected using a transmission electron microscope (TEM). The morphology of GO, rGO, GO + miRNA and rGO + miRNA was inspected using a JEOL JEM-1220 TEM (JEOL Ltd., Tokyo, Japan) at 80 KeV equipped with a Morada 11-megapixel camera (Olympus Corporation, Tokyo, Japan) (Figure 2A–D). Triplicate samples of GO and rGO were prepared for TEM by placing droplets of the hydrocolloid onto Formvar-coated copper grids (Agar Scientific Ltd., Stansted, UK) and air drying before TEM imaging.

#### 3.1.4. Dynamic Light Scattering (DLS)

Particle sizes were measured in a Zetasizer ZSP (Malvern Instrument Ltd., Worcestershire, UK) at 25 °C based on laser Doppler velocimetry and dynamic light scattering (DLS) techniques. Previously, the suspension was homogenized using an ultrasonication probe for a period of 30 min.

#### 3.1.5. Fourier Transform Infrared (FTIR) Spectroscopy

The FTIR spectra of the dry samples of GO, rGO and GO + antisense miRNAs (GO + miRNA), rGO + antisense miRNAs (rGO + miRNA) were recorded using a Nicolet 6700 FTIR spectroscope with diamond ATR pickup (Thermo Scientific, Wilmington, DE, USA). All samples were prepared by dropping 500 µL of sample suspension on a microscope glass and drying the suspension.

#### 3.1.6. Determination of miRNA Entrapment Efficiency

miRNA entrapment efficiency (EE) at graphene complexes was obtained from the determination of free miRNA concentration in GO + miRNA/rGO + miRNA supernatant recovered from centrifugation process. The concentrations of miRNA were added and a concentration of miRNA in GO + miRNA/rGO + miRNA supernatant were measured using a NanoDrop 2000 spectrophotometer (Thermo Scientific, Wilmington, DE, USA). The concentration of free miRNA was determined using Beer’s Law and calculations were done using the following equation: C = A/(ε × b) where A is the concentration of miRNA, ε is the extinction coefficient, and b is the path length of the cuvette. The sample was measured in triplicate.

The entrapment efficiency was calculated by using the following Equation (1):

(1) $$\mathrm{EE}=\frac{\mathrm{concentration}\;\mathrm{of}\;\mathrm{miRNA}\;\mathrm{added}-\mathrm{concentration}\;\mathrm{of}\;\mathrm{miRNA}\;\mathrm{in}\;\mathrm{supernatant}}{\mathrm{concentration}\;\mathrm{of}\;\mathrm{miRNA}\;\mathrm{added}}\times 100\%$$

#### 3.1.7. ζ-Potential Measurements

The ζ-potentials of GO, rGO, GO + antisense miRNAs and rGO + antisense miRNAs were measured by the laser dynamic scattering electrophoretic method, using Smoluchowski approximation with a Zetasizer Nano ZS90 (Malvern Instruments, Malvern, UK). Each sample was measured after stabilisation at 25 °C for 120 s. All measurements were performed in triplicate.

### 3.2. Cell Cultures and Treatments

All measurements were performed in triplicate.

GBM cell lines U87, U118, U251 and T98 and human foetal foreskin fibroblast HFFF2 were obtained from the American Type Culture Collection (Manassas, VA, USA) and maintained in Dulbecco’s modified Eagle supplemented with 10% foetal bovine serum (Sigma-Aldrich), 1% penicillin and streptomycin (Sigma-Aldrich) at 37 °C in a humidified atmosphere of 5% CO_2_/95% air in a NuAire DH AutoFlow CO_2_ Air-Jacketed Incubator (Plymouth, MN, USA).

### 3.3. Cell Morphology

A morphology investigation of U87, U118, U251 and T98 GBM and HFFF2 fibroblast after treatment with nanosheets of GO and rGO at concentrations 5.0, 10.0, 25.0, 50.0 and 100.00 µg/mL was conducted. Cells were placed on six-well plates (1 × 10^5^ cells per well). After 24 h of incubation with nanosheets of GO and rGO, cell morphology was recorded under an optical microscope (DM750; Leica Microsystems GmbH, Wetzlar, Germany) using the software package LAS EZ version 2.0. All measurements were performed in triplicate.

### 3.4. Cell Viability Assay

Cell viability was evaluated using a 2,3-bis-(2-methoxy-4-nitro-5-sulfophenyl)-2H-tetrazolium-5-carboxyanilide salt (XXT)-based cell viability assay kit (Life Technologies, Taastrup, Denmark). Cells U87, U118, U251, T98 and HFFF2 were incubated in 96-well plates (5 × 10^3^ cells per well) with hydrocolloids of nanoparticles of GO at concentrations 5.0, 10.0, 25.0, 50.0 and 100.00 µg/mL or with rGO at the same concentrations. In the subsequent step, XTT solution was added to each well and incubated for an additional 3 h at 37°C. The optical density (OD) of each well was recorded at 450 nm in a scanning multiwell spectrophotometer (Infinite M200, Tecan, Durham, NC, USA). Cell viability was expressed as a percentage (ODtest − ODblank) / (ODcontrol − ODblank), where ‘ODtest’ is the OD of cells exposed to GO and rGO, ‘ODcontrol’ is the OD of the control sample and ‘ODblank’ is the OD of wells without cancer cells.

Based on the obtained results for cell morphology and viability, the 100 µg/mL concentration was chosen to obtain the GO and rGO complexes functionalised with antisense miRNAs. The next step of cell investigation was followed for the complex of GO (100.00 µg/mL) functionalised with miRNA-124 and -137 and antisense miRNA-21, -221 and -222 (5 pmol/mL), rGO + antisense miRNA-21, -221 and -222 (100.00 µg/mL, 5 pmol/mL).

### 3.5. Cell Transfection

#### 3.5.1. Electroporation

For transfection by electroporation, the cells were cultured in a 75 cm^3^ flask (9 × 10^6^ cells). Next, the medium was discarded, and cells were washed two times with phosphate-buffered saline (PBS, pH 7.4; 10010023, Thermo Fisher Scientific, Waltham, MA, USA) and harvested. The cells were suspended into 200 µL of electroporation buffer (165-2676, Bio-Rad, Hercules, CA, USA) to a final cell concentration of 1 × 10^6^ and placed at 0.2 µm electroporation cuvette (165-2092, Bio-Rad, Hercules, CA, USA). Next, 2 µL of miRNA-124 and -137 and antisense miRNA-21, miRNA-221, miRNA-222 were introduced into the U87, U118, U251, T98 and HFFF2 cell lines. Electroporation was conducted under the following conditions: voltage 200 V, capacitance 900 µF, pulse duration 20 ms, resistance −20 Ω and single pulse using Gene Pulser Xcell Electroporation Systems (Bio-Rad, Hercules, CA, USA). After electroporation, the cells were cultivated in six-well plates (1 × 10^6^ cells per well) for 24 h. Next, the supernatant was removed, and the cells were washed with PBS and incubated in a fresh medium containing 10% FBS for 24 h. After 24 h, cell viability was investigated under the same condition as for GO and rGO.

#### 3.5.2. Transfection by Graphene Oxide and Reduced Graphene Oxide Complexes

For transfection, the cells were cultured in six-well plates (1 × 10^5^ cells per well) and the GO/rGO miRNA-124 and miRNA-137 and antisense miRNA-21, -221 and -222 at a concentration (GO/rGO_100 µg/mL_:miRNA_5 pmol/mL_) were added to each well and incubated for 24 h. After transfection, the cells were cultivated in six-well plates (1 × 10^5^ cells per well) for 24 h. Next, the supernatant was removed, and the cells were washed with PBS and incubated in a fresh medium containing 10% FBS for 24 h. After 24 h, the morphology of the cells and viability were investigated under the same condition as in steps 3.3 and 3.4 (Section 3.3 and Section 3.4).

#### 3.5.3. Cell Morphology after Transfection by Graphene Oxide and Reduced Graphene Oxide

The morphology investigation of U87, U118, U251 and T98 GBM, and HFFF2 fibroblast after transfection with GO + miRNA-124, -137 and antisense miRNA-21, -221 and -222 and rGO + miRNA-124 and -137 and antisense miRNA-21, -221, -222 at the same concentration (GO/rGO_100 µg/mL_:miRNA_5 pmol/mL_) was conducted. Cells after transfection and electroporation were placed on six-well plates (1 × 10^5^ cells per well). After 24 h, cell morphology was recorded under an optical microscope (DM750; Leica Microsystems GmbH, Wetzlar, Germany) using the software package LAS EZ version 2.0. All measurements were performed in triplicate.

#### 3.5.4. Cell Viability Assay after Transfection by Graphene Oxide and Reduced Graphene Oxide

Cell viability was evaluated using a 2,3-bis-(2-methoxy-4-nitro-5-sulfophenyl)-2H-tetrazolium-5-carboxyanilide salt (XXT)-based cell viability assay kit (Life Technologies, Taastrup, Denmark). Cells after transfection with GO + miRNA-124, -137 and antisense miRNA-21, -221 and -222 and rGO + miRNA-124, -137 and antisense miRNA-21, -221 and -222 at the same concentration (GO/rGO_100 µg/mL_:miRNA_5 pmol/mL_) were incubated in 96-well plates (5 × 10^3^ cells per well). Next, XTT solution was added to each well and incubated for an additional 3 h at 37°C. The OD of each well was recorded at 450 nm with a scanning multiwell spectrophotometer (Infinite M200, Tecan, Durham, NC, USA). Cell viability was expressed as a percentage (ODtest − ODblank)/(ODcontrol − ODblank), where ‘ODtest’ is the OD of cells exposed to GO and rGO, ‘ODcontrol’ is the OD of the control sample, and ‘ODblank’ is the OD of wells without cancer cells.

#### 3.5.5. Transfection Efficiency Evaluation

To evaluate the transfection efficiency of cells, 0.1 mg of FITC (F143, Thermo Fischer Scientific, Waltham, MA, USA) labelled the GO, rGO, miRNA sequences and GO + miRNA and rGO + miRNA sequences for overnight incubation at 4 °C, protected from light. Next, the miRNAs were introduced to the U87, U118, U251, T98 and HFFF2 cell lines by electroporation or transfection described in Section 3.5.1 and Section 3.5.2. The efficiency of the transfection technique was analysed by flow cytometry (FACSCalibur, Becton Dickinson, Franklin Lakes, NJ, USA) and confocal microscopy (IX 81 FV-1000 Olympus Corporation, Tokyo, Japan). Posttransfection image analysis in confocal mode, Nomarski interference contrast, and cell morphology observation were performed using FVIO-ASW version 1.7c (Olympus). Three-dimensional images were assembled from 30 optical sections. For flow cytometry, 8000 events were recorded per sample. Fluorescence emission intensity was measured using FL1 channels for FITC at Em = 530 nm using excitation at 488 nm. Histograms were generated using Flowing Software 2.5.1 (Perttu Terho, Turku, Finland).

### 3.6. Gene Expression

#### 3.6.1. Isolation of Total RNA

For the isolation of total RNA, U87, U118, U251, T98 and HFFF2 cells (1 × 10^5^ cells per well) were incubated for 24 h. Subsequently, the medium was removed, and GO + miRNA-124, -137 and antisense miRNA-21, -221 and -222 (GO_100 µg/mL_:miRNA_5 pmol/mL_) were added to the cells and incubated for an additional 24 h. Total RNA was isolated using a PureLink^®^ RNA Mini Kit (Ambion™ Life Technologies, Foster City, CA, USA). The resulting cell pellet was resuspended in a lysis buffer containing 1% 2-mercaptoethanol, and subsequently, the frozen metal balls were added to the probe and homogenised in a TissueLyser ball mill (Qiagen, Germantown, MD, USA) for 5 min at 50 Hz. The homogenate was centrifuged at 12,000× *g*. The supernatant containing total RNA was transferred into a new tube, and one volume of 70% ethanol was added into each volume of cell homogenate, following manufacturer’s instructions. Total RNA was eluted in 50 µL RNase-free water and stored at −80 °C. The isolated RNA was measured using a NanoDrop 2000 spectrophotometer (Thermo Scientific, Wilmington, DE, USA). The cDNA was synthesised with a cDNA High Capacity Reverse Transcription Kit (AppliedBiosystems, Foster City, CA, USA) to reverse-transcript the mRNA to cDNA using 2200 ng per reaction. The obtained cDNA was measured using a NanoDrop 2000 spectrophotometer and stored for further analysis at −20 °C.

#### 3.6.2. Real-Time PCR

The ∆∆Ct method was used to determine the expression of mRNA using real-time PCR (Equation (2)):

∆∆CT = ∆CT test sample − ∆CT calibrator sample(2)

The reaction was carried out using 48-well plates and the Luminaris Color HiGreen reagents qPCR Master Mix (Thermo Fisher Scientific); 100 ng of cDNA was used for each reaction. The following genes were examined: p21, p53, B-cell lymphoma 2 (Bcl-2), BAX and PCNA. The primers used for this procedure are presented in Table 1. Glyceraldehyde-3-phosphate dehydrogenase (GPDH) was used as the reference housekeeping gene. The reaction conditions were set as specified by the manufacturer, and each sample was analysed in duplicate. The procedure was conducted using a StepOnePlus™ Real-Time PCR System. qPCR results were normalised to the control (Log2FC = 0). Relative expression was calculated using the GPDH gene and control group (0).

### 3.7. Isolation of Total Protein

For protein analysis, glioma cells were treated with representative complexes GO + miRNAs. Cells not treated with nanoparticles were used as the control. Whole-cell protein extracts were prepared by suspending cells in ice-cold radioimmunoprecipitation assay (RIPA) buffer containing protease and phosphatase inhibitors (Sigma-Aldrich, St. Louis, MO, USA). The cells were incubated on ice for 30 min. (vortexing at 10 min. intervals) before being centrifuged (30 min; 14,000× *g*; 4 °C), and the supernatant was collected. Nuclear fraction was obtained by suspending cells in hypotonic buffer (20 mM Tris-HCl, pH 7.4; 10 mM NaCl; 3 mM MgCl_2_). Igepal CA-630 (Sigma-Aldrich) containing protease, and phosphatase inhibitors (Sigma-Aldrich) was added to a final concentration of 0.5%, and the solution was vortexed for 10 s. The pellet containing the nuclear fraction was resuspended in an ice-cold RIPA buffer containing protease, and phosphatase inhibitors and incubated on ice for 30 min. (vortexing at 10 min. intervals). The supernatant of the nuclear fraction homogenate was collected after centrifugation (30 min.; 14,000× *g*; 4 °C). Protein concentration was determined using a Bicinchoninic Acid Kit (Sigma-Aldrich).

#### Human Apoptosis Antibody Array

Analysis of apoptosis was performed using an antibody array (ab134001; Abcam, Cambridge, UK). The assay was performed in accordance with manufacturer’s instructions using lysates containing 200 μg/mL of total protein per membrane. Membranes were visualised using the Azure Biosystem C400 (Azure, Dublin, CA, USA) [64]. Table 2 presents the full array map. The results shown in Figure 17 were obtained by analysis in ImageJ. Results were normalised and compared to a dot control sample.

### 3.8. Statistical Analysis

Data were analysed using one-way analysis of variance (ANOVA) in StatPlus (AnalystSoft, Walnut, CA, USA). Differences between groups were tested using Tukey’s multiple-range tests. Differences at *p* < 0.05 were considered significant. All mean values were presented with standard deviations.

## 4. Conclusions

In this work, we have developed new complexes for effective nucleic acid delivery into GBM and fibroblast cell lines. GO and rGO complexes were synthesised, characterised and utilised to reduce viability and activate the programmed cell death pathway by apoptosis at GBM cell lines. The primary in vitro studies point to GO as a more stable and effective complex for selected miRNA sequence delivery. The results show that the extent of suppression of GBM cell viability due to the delivery of GO + antisense miRNA was more effective than transfection by electroporation (Table 3). Transfection by GO + antisense miRNA-21 activated apoptosis through two major types: intrinsic pathways, with increased expression of BAX, cytoC and Smac, and extrinsic pathways, where upregulation of caspase-8 was observed. Bidirectional activation of apoptosis was the result of the synergism of physical, chemical and biological features of GO and antisense miRNA-21. This indicates that GO + antisense miRNA-21 is a promising complex for delivering synthetic miRNA in GBM therapy.

## Figures and Tables

**Table 1:** Primer sequences for the investigated genes.

Target Gene	Forward Primer	Reverse Primer
p21	CCTCATCCCGTGTTCTCCTTT	GTACCACCCAGCGGACAAGT
p53	TTGCAATAGGTGTGCGTCAGA	AGTGCAGGCCAACTTGTTCAG
Bcl-2	GAAGAGCAAATGAGCCAAAC	AACCTATGCAATGGGATTGA
BAX	GATGCGTCCACCAAGAAGCT	CGGCCCCAGTTGAAGTTG
PCNA	AGGCACTCAAGGACCTCATCA	GAGTCCATGCTCTGCAGGTTT
GAPDH	TGCACCACCAACTGCTTAGC	GGCATGGACTGTGGTCATGAG

Abbreviations: Bcl-2: cell lymphoma 2, BAX: Bcl-2-associated X protein, PCNA: proliferating cell nuclear antigen, GPDH: glyceraldehyde-3-phosphate dehydrogenase.

**Table 2:** Apoptosis protein array map.

**Lin./Col.**	**C1**	**C2**	**C3**	**C4**	**C5**	**C6**	**C7**
L1	Pos	Pos	Neg	Neg	BLANK	BLANK	Bad
L2	Pos	Pos	Neg	Neg	BLANK	BLANK	Bad
L3	CD40	CD40L	cIAP-2	cytoC	DR6	Fas	FasL
L4	CD40	CD40L	cIAP-2	cytoC	DR6	Fas	FasL
L5	IGFBP-1	IGFBP-2	IGFBP-3	IGFBP-4	IGFBP-5	IGFBP-6	IGF-1sR
L6	IGFBP-1	IGFBP-2	IGFBP-3	IGFBP-4	IGFBP-5	IGFBP-6	IGF-1sR
L7	sTNF-R2	TNF-α	TNF-β	TRAILR-1	TRAILR-2	TRAILR-3	TRAILR-4
L8	sTNF-R2	TNF-α	TNF-β	TRAILR-1	TRAILR-2	TRAILR-3	TRAILR-4
**Lin./Col.**	**C8**	**C9**	**C10**	**C11**	**C12**	**C13**	**C14**
L1	Bax	Bcl-2	Bcl-w	BID	BIM	Caspase 3	Caspase 8
L2	Bax	Bcl-2	Bcl-w	BID	BIM	Caspase 3	Caspase 8
L3	BLANK	HSP27	HSP60	HSP70	HTRA	IGF-I	IGF-II
L4	BLANK	HSP27	HSP60	HSP70	HTRA	IGF-I	IGF-II
L5	Livin	p21	p27	p53	SMAC	Survivin	sTNF-R1
L6	Livin	p21	p27	p53	SMAC	Survivin	sTNF-R1
L7	XIAP	BLANK	BLANK	Neg	Neg	Neg	Pos
L8	XIAP	BLANK	BLANK	Neg	Neg	Neg	Pos

Abbreviations: Pos—positive control, Bad—Bcl-2-associated death promoter, BAX—BCL2 Associated X Apoptosis Regulator, Bcl-2—B-cell lymphoma 2, Bcl-w—Bcl-2-like protein 2, BID—BH3 Interacting Domain Death Agonist, BIM—Bcl-2-like protein 11, Caps3—Caspase 3, Caps8—Caspase 8, CD40—CD-40 protein, CD40L—CD 40 protein ligand, cIAP-2—Cellular inhibitor of apoptosis 2, cytoC—Cytochrome C, DR6—Death receptor 6, Fas—Fas cell surface death receptor, FasL—Fas ligand (TNF superfamily, member 6), HSP27—Heat shock protein 27, Hsp60—Heat shock protein 60, Hsp70—Heat shock protein 70, HTRA—serine protease HtrA, IGF-I—Insulin like growth factor level, IGF-II—Insulin like growth factor 2, IGFBP-1—Insulin-like growth factor-binding protein 1, IGFBP-2—Insulin-like growth factor-binding protein 2, IGFBP-3—Insulin-like growth factor-binding protein 3, IGFBP-4—Insulin-like growth factor-binding protein 4, IGFBP-5—Insulin-like growth factor-binding protein 5, IGFBP-6—Insulin-like growth factor-binding protein 6, IGF-1sR—Insulin like growth factor 1 receptor, Livin—baculoviral IAP repeat (BIR) domain, p21—Cyclin-dependent kinase inhibitor 1, p27—Cyclin-dependent kinase inhibitor, p53—protein p53, SMAC—diablo IAP-binding mitochondrial protein, Survivin—Baculoviral IAP repeat containing 5, sTNF-R1—TNF receptor superfamily member 1A, sTNF-R2—TNF receptor superfamily member 1B, TNF-α—Tumour necrosis factor alpha-like, TNF-β—Tumour necrosis factor beta-like, TRAILR-1—TNF receptor superfamily member 1, TRAILR-2—TNF receptor superfamily member 2, TRAILR-3—TNF receptor superfamily member 3, TRAILR-4—TNF receptor superfamily member 4, XIAP—X-linked inhibitor of apoptosis protein.

**Table 3:** Summary table.

Nanostructure		Cell Morphology	Cell Viability GBM U87, U118, U251, T98	Gene and Protein Expression
Complexes of GO + miRNA-21		Effect the cell structure, cell deformation, shortened tentacles	Reduce cell viability of U87, U118, U251 and T98 GBM cell line	Increase gene expression level of p21, p53, BAX Downregulate PCNA gene expression level Increased the protein expression level of BAX, cytoC, Smac and caspase -8
Complexes of rGO + antisense miRNA-21		Effect the cell structure, minor cell deformation	No cytotoxic effect against U87, U118, U251 and T98 GBM cell line	-
miRNA	Antisense miRNA-21	Effect the cell structure, cell deformation, shortened tentacles	Reduce cell viability of U87, U118, U251 and T98 GBM cell line	Increase expression level of p21, p53, BAX Downregulate PCNA expression level

## Data Availability

Not applicable.

## Supplementary Materials

The following are available online, Figure S1. Morphology of U87 glioblastoma cells; Figure S2. Morphology of U118 glioblastoma cells; Figure S3. Morphology of U251 glioblastoma cells; Figure S4. Morphology of T98 glioblastoma cells; Figure S5. Morphology of HFFF-2 human fibroblast cell line.

## Sample Availability

Samples of the GO+ miRNA -21 are available from the authors.
