# Supplementary material for: MicroRNA Delivery by Graphene-Based Complexes into Glioblastoma Cells
Source: Molecules. 2021 Sep 25;26(19):5804. doi: 10.3390/molecules26195804 (PMC8510190; doi:10.3390/molecules26195804)
Supplement: Supplementary file 1 [file molecules-26-05804-s001.zip › molecules-1377051-supplementary.pdf]

Supplementary materials

MicroRNA delivery by graphene-based complexes into glioblastoma cells

Kutwin M.[1]*, Sosnowska E. M.[1], Strojny-Cieślak B.[1], Jaworski S.[1], Trzaskowski M.[2], Wierzbicki M.[1], Chwalibog A.[3], Sawosz E.[1]

[1] Department of Nanobiotechnology, Institute of Biology, Warsaw University of Life Sciences, 02-786 Warsaw, Poland; marta_kutwin@sggw.edu.pl (M.K.); malwina_sosnowska@sggw.edu.pl (M.E.S.); barbara_strojny@sggw.edu.pl (B.S), slawomir_jaworski@sggw.pl (S.J.); mateusz_wierzbicki@sggw.edu.pl (M.W.); ewa_sawosz@sggw.edu.pl (E.S.);

[2] Centre for Advanced Materials and Technologies CEZAMAT, Warsaw University of Technology, 02-822 Warsaw, Poland; m.trzaskowski@cezamat.pl

[3] Department of Veterinary and Animal Sciences, Faculty of Health and Medical Sciences, University of Copenhagen, 1870 Frederiksberg, Denmark; ach@sund.ku.dk

* Correspondence: marta_kutwin@sggw.edu.pl; Tel.:+48-225936671

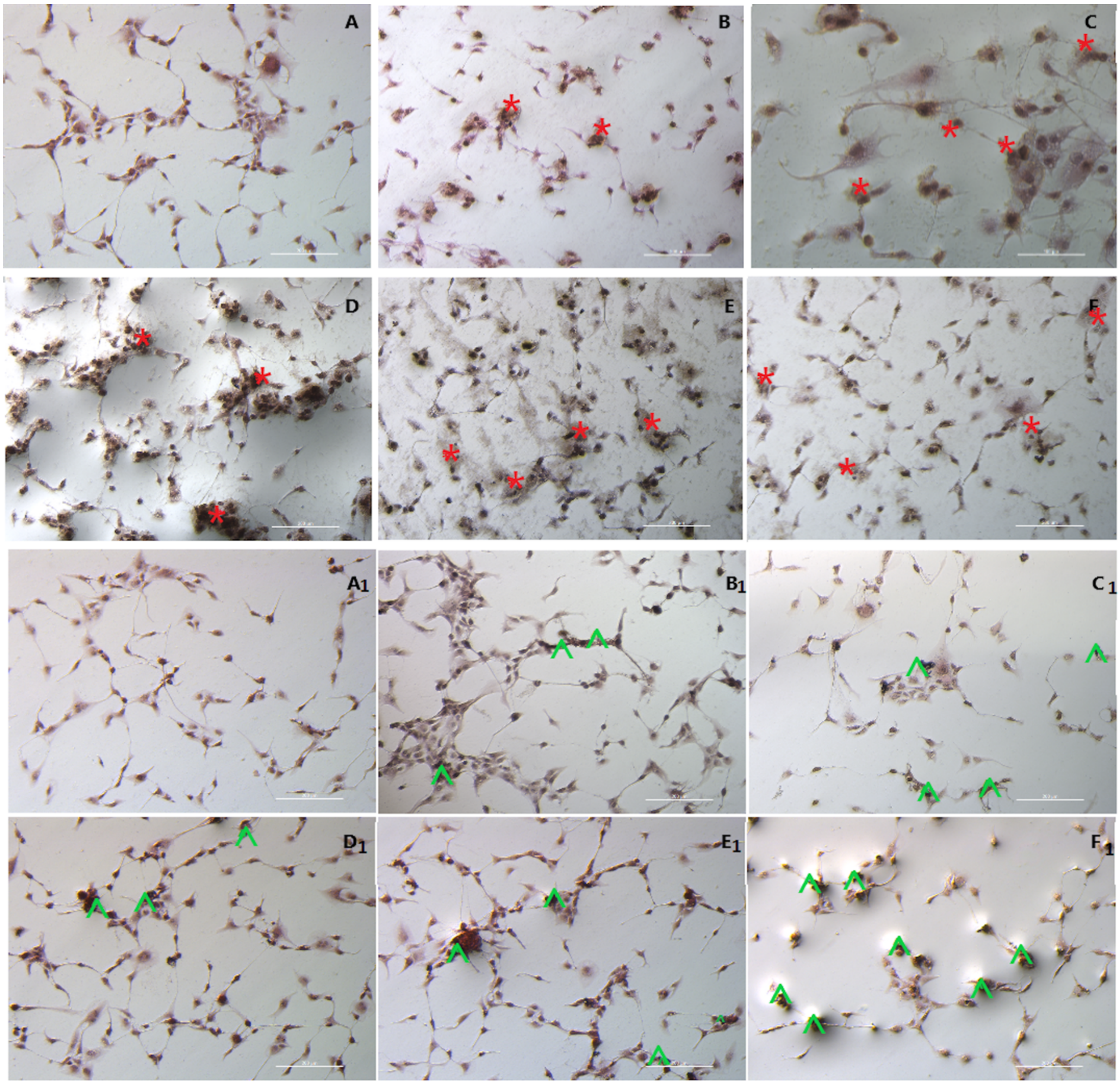

Figure S1. Morphology of U87 glioblastoma cells. (A, A1) untreated cells (control group), cells treated with graphene oxide at concentration: 5 µg/ml (B), 10 µg/ml (C), 25 µg/ml (D), 50 µg/ml (E) and 100 µg/ml (F) and reduced graphene oxide at concentration: 5 µg/ml (B1), 10 µg/ml (C1), 25 µg/ml (D1), 50 µg/ml (E1) and 100 µg/ml (F1). Red *: GO at cell membrane. Green *: rGO at cell membrane. Light optical microscopy. Scale bars: 100 µm. (A–F). Abbreviations: GO: graphene oxide, rGO: reduced graphene oxide.

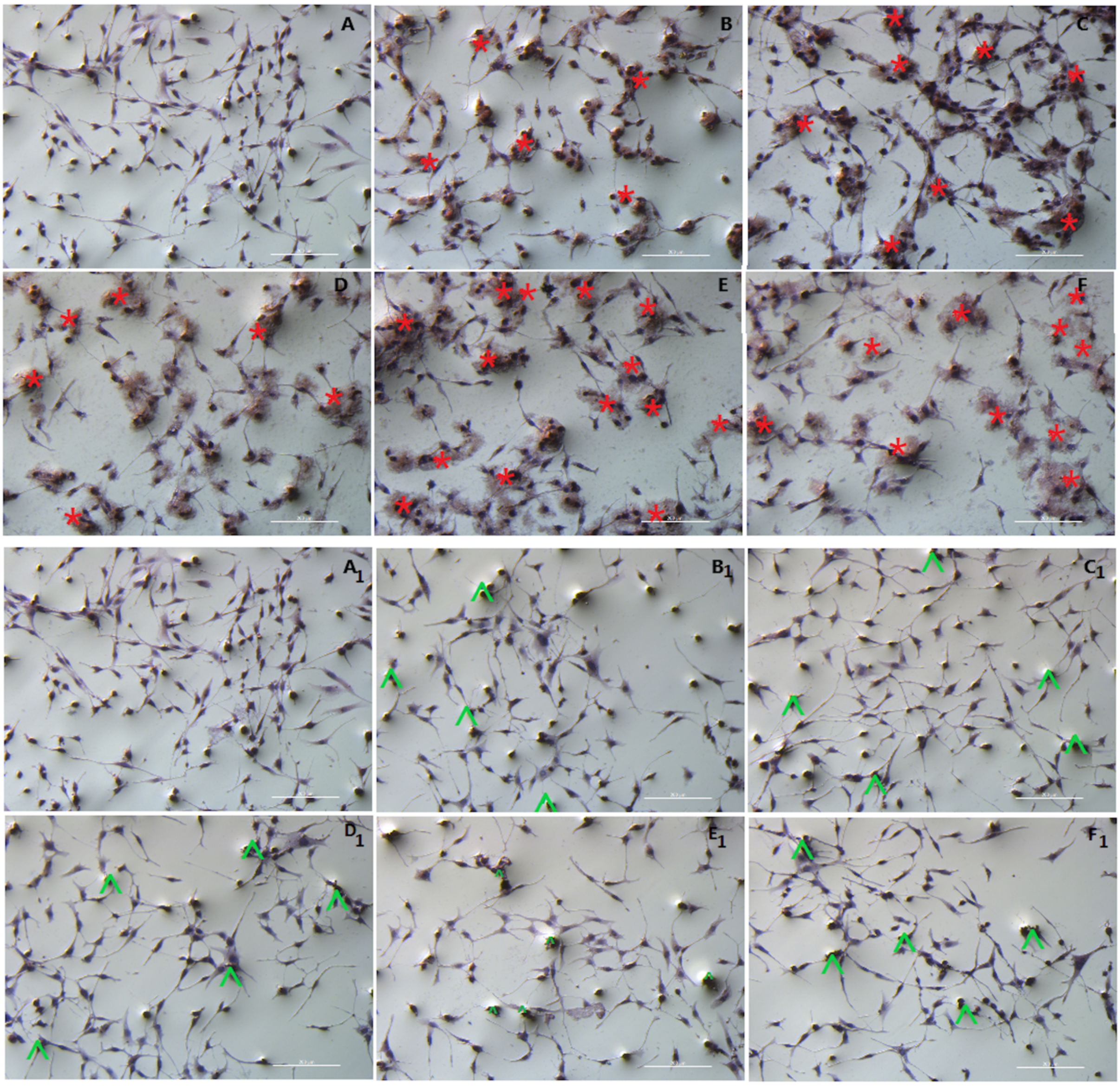

Figure S2. Morphology of U118 glioblastoma cells. (A, A1) untreated cells (control group), cells treated with graphene oxide at concentration: 5 µg/ml (B), 10 µg/ml (C), 25 µg/ml (D), 50 µg/ml (E) and 100 µg/ml (F) and reduced graphene oxide at concentration: 5 µg/ml (B1), 10 µg/ml (C1), 25 µg/ml (D1), 50 µg/ml (E1) and 100 µg/ml (F1). Red *: GO at cell membrane. Green *: rGO at cell membrane. Light optical microscopy. Scale bars: 100 µm. (A–F). Abbreviations: GO: graphene oxide, rGO: reduced graphene oxide.

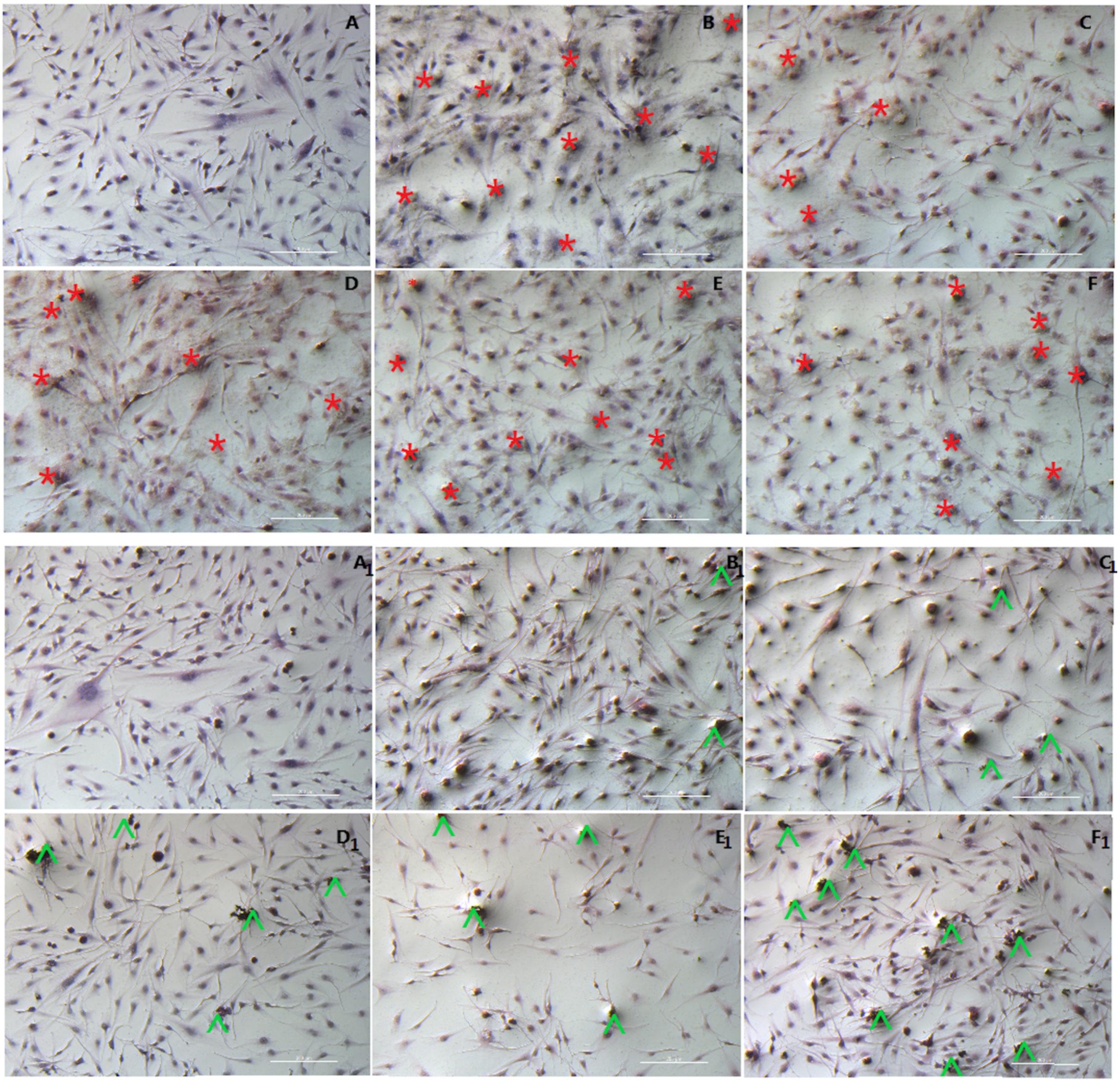

Figure S3. Morphology of U251 glioblastoma cells. (A, A1) untreated cells (control group), cells treated with graphene oxide at concentration: 5 µg/ml (B), 10 µg/ml (C), 25 µg/ml (D), 50 µg/ml (E) and 100 µg/ml (F) and reduced graphene oxide at concentration: 5 µg/ml (B1), 10 µg/ml (C1), 25 µg/ml (D1), 50 µg/ml (E1) and 100 µg/ml (F1). Red *: GO at cell membrane. Green *: rGO at cell membrane. Light optical microscopy. Scale bars: 100 µm. (A–F). Abbreviations: GO: graphene oxide, rGO: reduced graphene oxide.

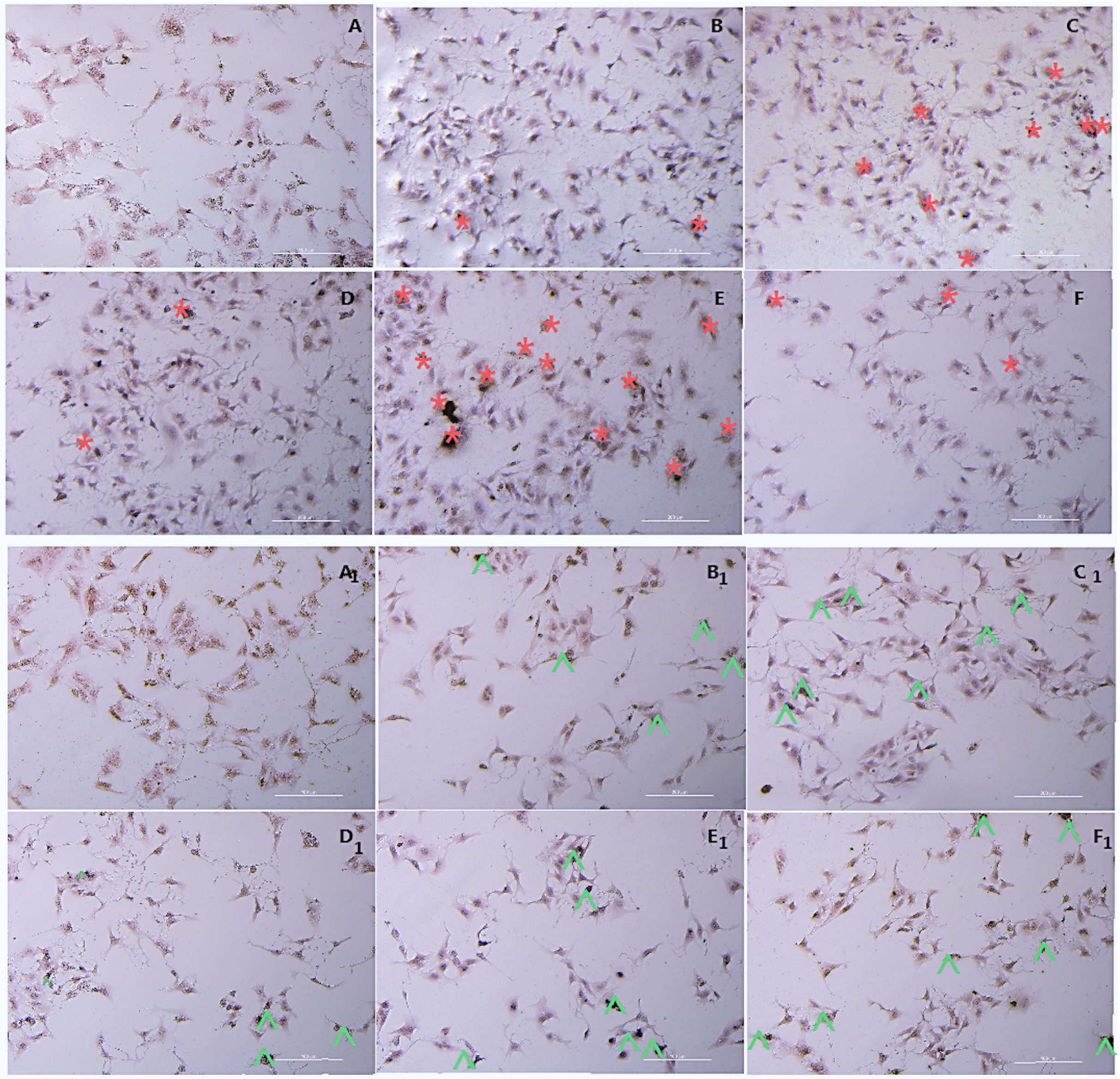

Figure S4. Morphology of T98 glioblastoma cells. (A, A1) untreated cells (control group), cells treated with graphene oxide at concentration: 5 µg/ml (B), 10 µg/ml (C), 25 µg/ml (D), 50 µg/ml (E) and 100 µg/ml (F) and reduced graphene oxide at concentration: 5 µg/ml (B1), 10 µg/ml (C1), 25 µg/ml (D1), 50 µg/ml (E1) and 100 µg/ml (F1). Red *: GO at cell membrane. Green *: rGO at cell membrane. Light optical microscopy. Scale bars: 100 µm. (A–F). Abbreviations: GO: graphene oxide, rGO: reduced graphene oxide.

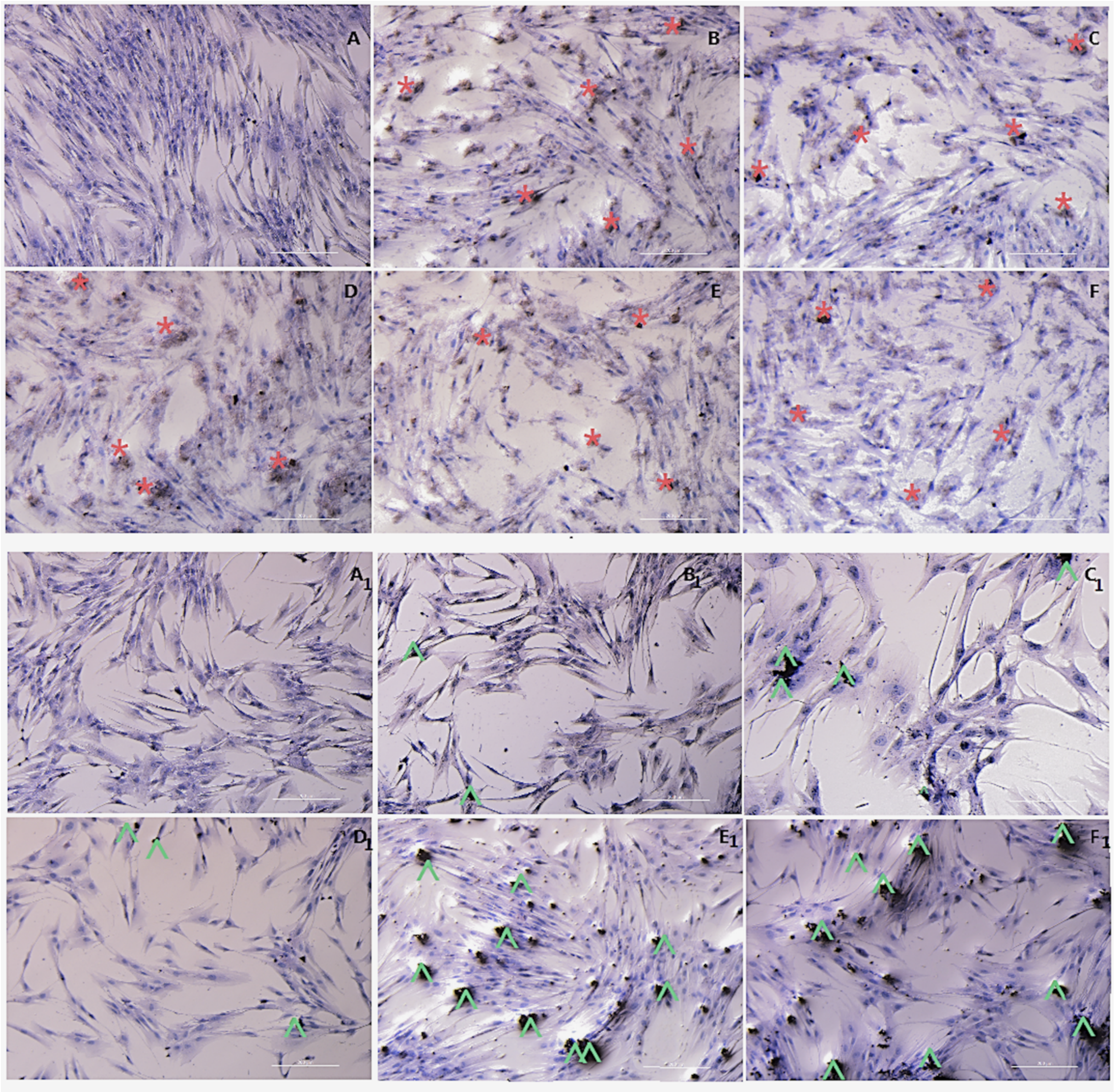

Figure S5. Morphology of HFFF-2 human fibroblast cell line . (A, A1) untreated cells (control group), cells treated with graphene oxide at concentration: 5 µg/ml (B), 10 µg/ml (C), 25 µg/ml (D), 50 µg/ml (E) and 100 µg/ml (F) and reduced graphene oxide at concentration: 5 µg/ml (B1), 10 µg/ml (C1), 25 µg/ml (D1), 50 µg/ml (E1) and 100 µg/ml (F1). Red *: GO at cell membrane. Green *: rGO at cell membrane. Light optical microscopy. Scale bars: 100 µm. (A–F). Abbreviations: GO: graphene oxide, rGO: reduced graphene oxide.
